# Supplementary material for: Deploying green hydrogen to decarbonize China’s coal chemical sector
Source: Nat Commun. 2023 Dec 7;14:8104. doi: 10.1038/s41467-023-43540-4 (PMC10703803; doi:10.1038/s41467-023-43540-4)
Supplement: Supplementary file 1 — Supplementary Information [file 41467_2023_43540_MOESM1_ESM.pdf]

SUPPLEMENTARY INFORMATION

Deploying Green Hydrogen to Decarbonize China's Coal Chemical Sector

Yang Guo[1,*], Liqun Peng[1], Jinping Tian[2], Denise L. Mauzerall[1,3,*]

[1] Princeton School of Public and International Affairs, Princeton University, NJ 08544, USA

[2] School of Environment, Tsinghua University, Beijing 100084, China

[3] Department of Civil and Environmental Engineering, Princeton University, NJ 08544, USA

* Correspondence: yangguo@princeton.edu, mauzerall@princeton.edu

These authors jointly supervised this work: Yang Guo, Denise L. Mauzerall

This file includes:

Supplementary Figures 1-5,

Supplementary Tables 1-25,

and Supplementary References.

Supplementary Figures

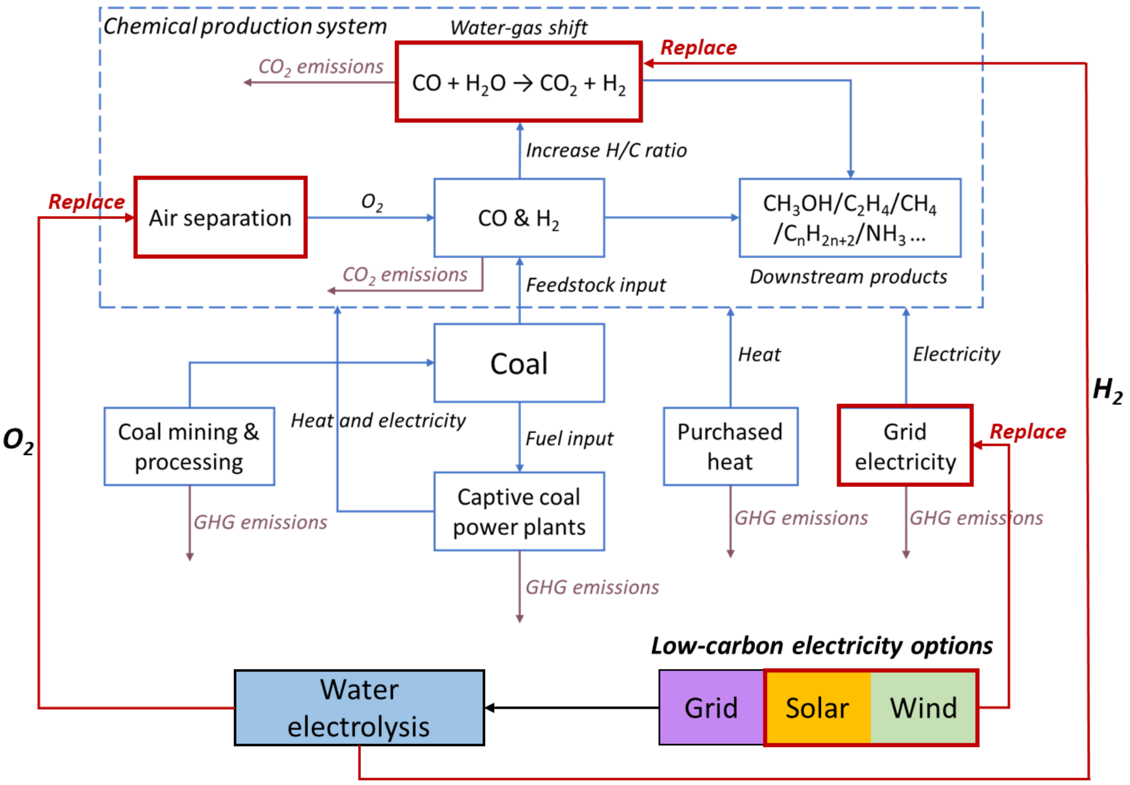

Supplementary Figure 1 Coal chemical production systems and low-carbon measures. Renewable or grid electricity can drive water electrolysis to generate H_2 and O_2 to replace H_2 from coal gasification and the water-gas shift reaction and O_2 from coal-driven air separation. Renewable electricity can also replace purchased grid electricity. GHG = greenhouse gas.

	Coke	Calcium carbide	Ammonia	Methanol	Oil (DL)	Oil (IDL)	Natural gas	Olefin	Ethylene glycol
Beijing									
Tianjin	0.27		0.50	1.3					
Hebei	7.4		7.0	5.6					1.2
Shanxi	16	1.1	14	14		4.1			2.2
Inner Mongolia	6.5	32	3.3	39	3.9	5.5	7.3	33	6.7
Liaoning	3.5	0.18	2.4	0.74					
Jilin	0.56		1.2	0.049					
Heilongjiang	1.6	0.067	1.7	2.3					
Shanghai	0.83			5.2					
Jiangsu	2.0		8.9	4.4					
Zhejiang	0.33		2.0	0.82					
Anhui	1.9	0.10	9.1	3.9				6.3	2.2
Fujian	0.34		2.2	1.6					
Jiangxi	1.1	0.86	0.22	0.10					
Shandong	4.8		19	34					4.2
Henan	2.8	1.9	17	20					5.8
Hubei	1.2	0.93	13	2.4					1.1
Hunan	0.92	0.33	2.7	0.28					
Guangdong	0.91		0.018						
Guangxi	1.2		2.7	0.82					
Hainan			2.0	8.0					
Chongqing	0.43	0.57	5.5	11					
Sichuan	1.6	3.2	10	1.8					
Guizhou	0.66		6.2	2.4					1.7
Yunnan	1.7	1.9	6.1	2.8					
Tibet									
Shaanxi	7.5	9.4	5.0	24		5.9		22	
Gansu	0.79	2.7	1.4	1.9					
Qinghai	0.28	0.18	0.038	0.81				9.3	
Ningxia	1.4	13	1.6	28		14		20	
Xinjiang	3.4	19	5.4	6.2			14	6.1	5.3

0 — 39 ($MtCO_2eq$)

Supplementary Figure 2 Provincial onsite greenhouse gas emissions from China's coal chemical production in 2020. Onsite emissions include those from chemical processes and onsite fuel combustion. DL = direct liquefaction and IDL = indirect liquefaction. Source data are provided as a Source Data file.

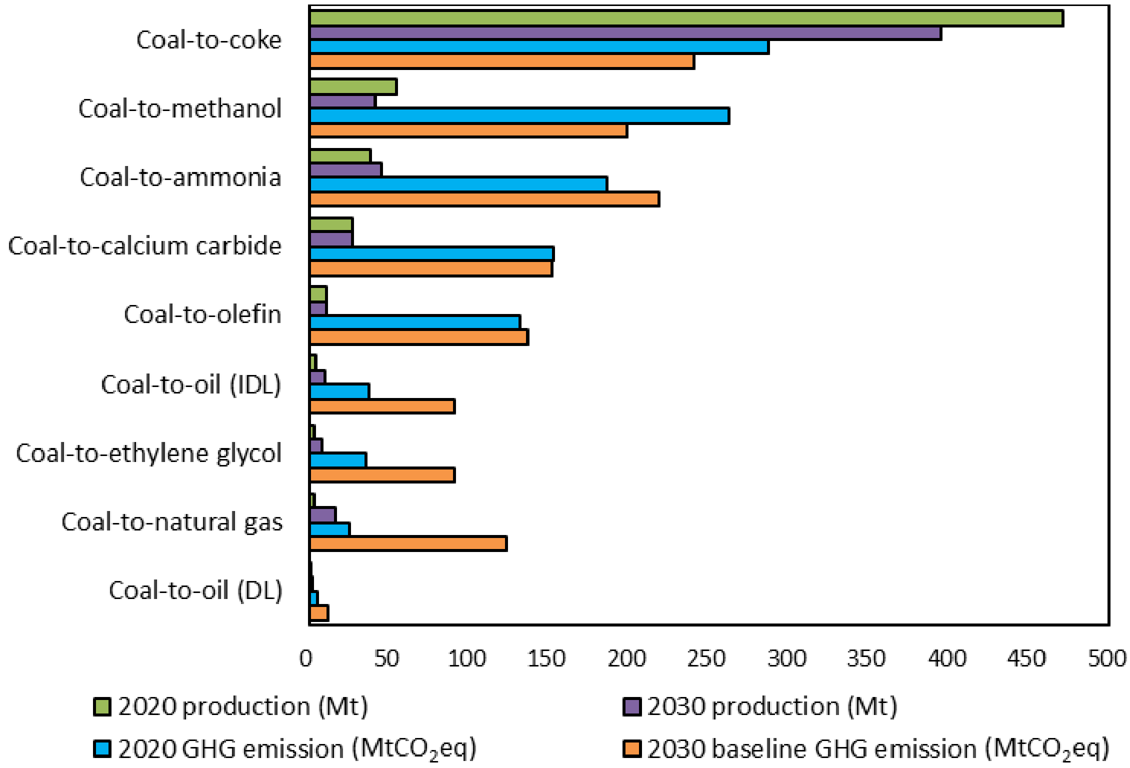

Supplementary Figure 3 China's coal chemical production and its greenhouse gas emissions in 2020 and the 2030 baseline scenario. DL = direct liquefaction and IDL = indirect liquefaction. GHG = greenhouse gas. Source data are provided as a Source Data file.

	Coke	Calcium carbide	Ammonia	Methanol	Oil (DL)	Oil (IDL)	Natural gas	Olefin	Ethylene glycol
Beijing									
Tianjin	0.23		0.59	1.0					
Hebei	6.2		8.3	4.3					2.0
Shanxi	13	1.1	16	11		7.3			3.6
Inner Mongolia	5.4	32	3.9	29	9.3	22	40	29	20
Liaoning	2.9	0.18	2.8	0.56			17		
Jilin	0.47		1.4	0.037					
Heilongjiang	1.4	0.067	1.9	1.7					
Shanghai	0.69			3.9					
Jiangsu	1.7		10	3.3					
Zhejiang	0.27		2.3	0.62					
Anhui	1.6	0.10	11	3.0				5.6	3.6
Fujian	0.29		2.6	1.2					
Jiangxi	0.88	0.86	0.26	0.078					
Shandong	4.1		23	26					6.8
Henan	2.4	1.9	20	15					9.6
Hubei	1.0	0.93	15	1.8					1.8
Hunan	0.78	0.33	3.2	0.21					
Guangdong	0.77		0.022						
Guangxi	1.0		3.1	0.62					
Hainan			2.4	6.1					
Chongqing	0.36	0.57	6.5	8.2					
Sichuan	1.4	3.2	12	1.4					
Guizhou	0.55		7.3	1.8					2.7
Yunnan	1.4	1.9	7.2	2.1					
Tibet									
Shaanxi	6.3	9.4	5.9	18		10		25	19
Gansu	0.66	2.7	1.6	1.4					
Qinghai	0.23	0.18	0.044	0.62				14	
Ningxia	1.2	13	1.9	21		25		22	
Xinjiang	2.9	19	6.3	4.7		6.3	48	5.5	8.7

0 — 48 ($MtCO_2eq$)

Supplementary Figure 4 Provincial onsite greenhouse gas emissions from China's coal chemical production in the 2030 baseline scenario. Onsite emissions include those from chemical processes and onsite fuel combustion. DL = direct liquefaction and IDL = indirect liquefaction. Source data are provided as a Source Data file.

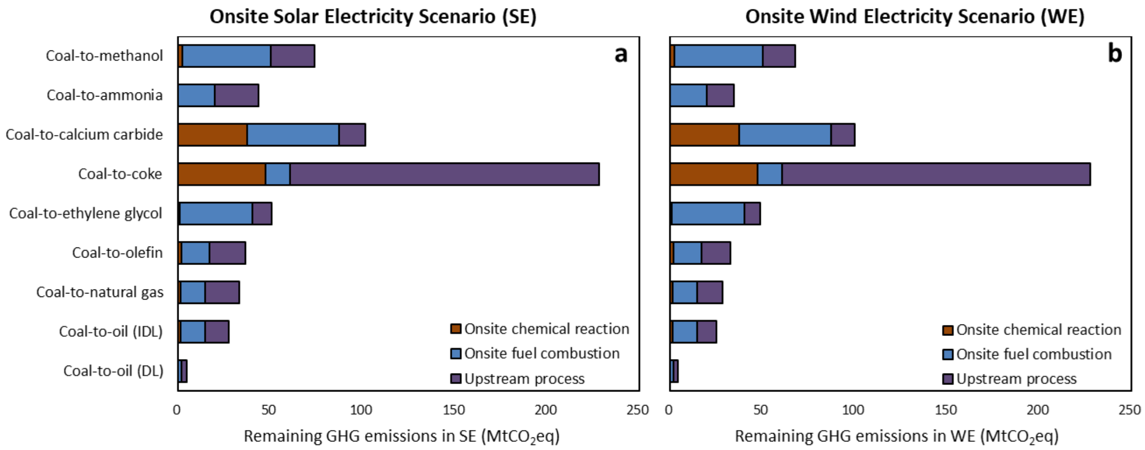

Supplementary Figure 5 Remaining greenhouse gas emissions from coal chemical production in the Onsite Solar Electricity (SE) and Onsite Wind Electricity (WE) scenarios. a, Onsite Solar Electricity scenario; and b, Onsite Wind Electricity scenario. GHG = greenhouse gas. DL = direct liquefaction and IDL = indirect liquefaction. Source data are provided as a Source Data file.

Supplementary Note: Methods

Supplementary Tables and related calculations are presented in the order of the Results in the main text: 1) GHG emission accounting, 2) GHG mitigation modeling, and 3) Cost-benefit analyses. We provide our modeling framework in Figure 6.

GHG Emissions Accounting: We track GHG emissions from coal chemical production using 2020 chemical production quantities *(Supplementary Tables 1-3)* and 2030 projected chemical production quantities *(Supplementary Tables 4-6)* coupled with GHG emission factors of coal chemical production in 2020 *(Supplementary Tables 7-10)* and in the 2030 baseline scenario *(Supplementary Table 11)*, respectively.

Second, we model the GHG mitigation potential of four alternative scenarios based on parametrizations of chemical reactions and supporting industrial processes, detailed in *Supplementary Tables 12-19*. Specifically, we use projected 2030 provincial production of coal chemicals *(Supplementary Table 6)* and demand per tonne of coal chemicals for electrolytic H_2 and O_2 *(Supplementary Table 13)* and for grid electricity *(Supplementary Table 8)* to estimate the provincial demand for electrolytic H_2 and O_2 and grid electricity (that can be replaced by onsite renewable electricity). Then we characterize GHG emission changes by provincial region and by individual process using these provincial demands with GHG emission factors of all major chemical reactions required the production of the eight coal chemicals analyzed *(Supplementary Tables 13)*, coal production, 2030 grid electricity generation *(Supplementary Table 9)*, air separation, coal gasification, electrolyzer and battery manufacturing, H_2 leakage *(Supplementary Table 15)*, and provincial solar/wind electricity generation *(Supplementary Table 16)*. We present provincial GHG emission changes of four alternative scenarios relative to the baseline scenario by individual process in *Supplementary Table 14* and we detail how to attribute GHG emission changes of individual processes to provincial regions in *Supplementary Tables 14 and 17*. We present national average GHG emission factors of

coal chemical production in the SE/WE scenarios in *Supplementary Table 18* and remaining GHG emissions in the SE/WE scenarios in *Supplementary Table 19*.

Third, we collect or estimate the cost parameters for coal chemical production and water electrolysis in *Supplementary Table 20*. We estimate the cost parameters for provincial solar/wind electricity generation in *Supplementary Table 21*. We then use these cost parameters with the provincial demands for electrolytic H_2 and O_2 and additional grid/onsite renewable electricity derived above to estimate provincial cost changes by individual processes in the SE/WE scenarios *(Supplementary Table 22)*.

In addition, we derive the provincial options for either solar or wind-based water electrolysis to achieve the maximum GHG mitigation or minimum cost of national coal chemical production *(Supplementary Table 23)* to provide provincial-level policy implications. We also analyze the GHG mitigation and economic benefits of selling excess green O_2 *(Supplementary Table 24)* and the land area needed for renewables in the SE/WE scenarios *(Supplementary Table 25)*.

Supplementary Tables

Supplementary Table 1 Provincial production of traditional coal chemicals in 2020

Provincial region	Coal-to-coke in 2020 (Mt)[1]	Coal-to-calcium carbide in 2020 (Mt)	Coal-to-ammonia in 2020 (Mt)	Coal-to-methanol in 2020 (Mt)	2018 ratios of provincial coal-to-calcium carbide production to total productoin[2]	2015 ratios of provincial coal-to-ammonia production to total production[3,4]	2015 ratios of provincial coal-to-methanol production to total production[3,4]
Beijing	0.00	0.00	0.00	0.00	0.0%	0.0%	0.0%
Tianjin	1.75	0.00	0.13	0.33	0.0%	0.3%	0.6%
Hebei	48.26	0.00	1.79	1.37	0.0%	4.7%	2.5%
Shanxi	104.94	0.35	3.47	3.53	1.3%	9.1%	6.5%
Inner Mongolia	42.23	10.18	0.85	9.50	36.9%	2.2%	17.4%
Liaoning	22.97	0.06	0.60	0.18	0.2%	1.6%	0.3%
Jilin	3.69	0.00	0.31	0.01	0.0%	0.8%	0.0%
Heilongjiang	10.63	0.02	0.42	0.56	0.1%	1.1%	1.0%
Shanghai	5.41	0.00	0.00	1.26	0.0%	0.0%	2.3%
Jiangsu	13.13	0.00	2.27	1.07	0.0%	5.9%	2.0%
Zhejiang	2.13	0.00	0.50	0.20	0.0%	1.3%	0.4%
Anhui	12.28	0.03	2.31	0.96	0.1%	6.1%	1.8%
Fujian	2.23	0.00	0.57	0.40	0.0%	1.5%	0.7%
Jiangxi	6.89	0.27	0.06	0.03	1.0%	0.1%	0.0%
Shandong	31.63	0.00	4.94	8.32	0.0%	12.9%	15.2%
Henan	18.48	0.59	4.30	4.90	2.1%	11.3%	9.0%

Hubei	8.01	0.29	3.27	0.58	1.1%	8.5%	1.1%
Hunan	6.04	0.10	0.69	0.07	0.4%	1.8%	0.1%
Guangdong	5.97	0.00	0.00	0.00	0.0%	0.0%	0.0%
Guangxi	8.12	0.00	0.68	0.20	0.0%	1.8%	0.4%
Hainan	0.00	0.00	0.51	1.95	0.0%	1.3%	3.6%
Chongqing	2.80	0.18	1.40	2.63	0.7%	3.7%	4.8%
Sichuan	10.74	1.03	2.58	0.44	3.7%	6.8%	0.8%
Guizhou	4.28	0.00	1.58	0.57	0.0%	4.1%	1.1%
Yunnan	10.93	0.60	1.57	0.68	2.2%	4.1%	1.2%
Tibet	0.00	0.00	0.00	0.00	0.0%	0.0%	0.0%
Shaanxi	48.97	2.96	1.27	5.83	10.8%	3.3%	10.7%
Gansu	5.17	0.85	0.35	0.46	3.1%	0.9%	0.8%
Qinghai	1.83	0.06	0.01	0.20	0.2%	0.0%	0.4%
Ningxia	9.21	3.99	0.42	6.84	14.5%	1.1%	12.5%
Xinjiang	22.47	6.02	1.37	1.51	21.8%	3.6%	2.8%
Total	471.16	27.58[5]	38.22[1,6]	54.57[7,8]	100%	100%	100%

Note: We obtain the 2020 provincial production of coal-to-coke directly from national statistics[1]. We collect the 2020 national production of coal-to-calcium carbide[5], coal-to-ammonia[1,6], and coal-to-methanol[7,8], and then use up-to-date distribution patterns of provincial production in the table to allocate the national production to provincial regions.

Supplementary Table 2 Provincial production of modern coal chemicals in 2020

Provincial region	Coal-to-oil (direct liquefaction, DL) in 2020 (Mt)	Coal-to-oil (indirect liquefaction, IDL) in 2020 (Mt)	Coal-to-natural gas (NG) in 2020 (Mt)	Coal-to-olefin in 2020 (Mt)	Coal-to-ethylene glycol in 2020 (Mt)	2020 ratios of provincial coal-to-oil (IDL) capacity to total[9]	2020 ratios of provincial coal-to-NG capacity to total[9]	2020 ratios of provincial coal-to-olefin capacity to total[9]	2020 ratios of provincial coal-to-ethylene glycol capacity to total[9]
Hebei	0.00	0.00	0.00	0.00	0.12	0.0%	0.0%	0.0%	4.0%
Shanxi	0.00	0.59	0.00	0.00	0.22	13.9%	0.0%	0.0%	7.3%
Inner Mongolia	0.97	0.78	1.14	3.66	0.66	18.4%	33.9%	33.9%	21.9%
Anhui	0.00	0.00	0.00	0.71	0.22	0.0%	0.0%	6.5%	7.3%
Shandong	0.00	0.00	0.00	0.00	0.41	0.0%	0.0%	0.0%	13.7%
Henan	0.00	0.00	0.00	0.00	0.58	0.0%	0.0%	0.0%	19.2%
Hubei	0.00	0.00	0.00	0.00	0.11	0.0%	0.0%	0.0%	3.7%
Guizhou	0.00	0.00	0.00	0.00	0.16	0.0%	0.0%	0.0%	5.5%
Shaanxi	0.00	0.84	0.00	2.50	0.00	19.8%	0.0%	23.1%	0.0%
Qinghai	0.00	0.00	0.00	1.04	0.00	0.0%	0.0%	9.6%	0.0%
Ningxia	0.00	2.03	0.00	2.22	0.00	47.9%	0.0%	20.5%	0.0%
Xinjiang	0.00	0.00	2.23	0.69	0.52	0.0%	66.1%	6.3%	17.4%
Total	0.97[10]	4.25[10]	3.37[10]	10.80[10]	3.00[10]	100%	100%	100%	100%

Note: We collect the 2020 national production of coal-to-oil (from direct and indirect coal liquefaction), coal-to-natural gas, coal-to-olefin, and coal-to-ethylene glycol[10], and then allocate the national production to provincial regions based on the ratios of 2020 provincial production capacities to national capacity listed in the table (totaling up capacities of individual projects in Supplementary Table 3).

Supplementary Table 3 Modern coal chemical projects in China in 2020[9]

No.	Project type	Project name	Capacity	Location	Status
1	Coal-to-oil (DL)	Shen'hua E'er'duo'si Direct Phase I	1.08 (Mt yr^{-1})	Inner Mongolia	In operation
2	Coal-to-oil (IDL)	Shen'hua Indirect	0.18 (Mt yr^{-1})	Inner Mongolia	In operation
3	Coal-to-oil (IDL)	Yi'tai E'er'duo'si Phase I	0.16 (Mt yr^{-1})	Inner Mongolia	In operation
4	Coal-to-oil (IDL)	Lu'an Chang'zhi	0.16 (Mt yr^{-1})	Shanxi	In operation
5	Coal-to-oil (IDL)	Yan'kuang Yu'lin	1.10 (Mt yr^{-1})	Shaanxi	In operation
6	Coal-to-oil (IDL)	Yan'kuang Yu'lin	0.10 (Mt yr^{-1})	Shaanxi	In operation
7	Coal-to-oil (IDL)	Ning'dong	4.00 (Mt yr^{-1})	Ningxia	In operation
8	Coal-to-oil (IDL)	Yi'tai Hang'jin'qi	1.20 (Mt yr^{-1})	Inner Mongolia	In operation
9	Coal-to-oil (IDL)	Lu'an Chang'zhi Integrated	1.00 (Mt yr^{-1})	Shanxi	In operation
10	Coal-to-oil (IDL)	Yan'chang Yu'lin	0.45 (Mt yr^{-1})	Shaanxi	In operation
11	Coal-to-oil (DL)	Shen'hua E'er'duo'si Phase II	1.50 (Mt yr^{-1})	Inner Mongolia	In planning
12	Coal-to-oil (IDL)	Yi'tai E'er'duo'si Phase II	2.00 (Mt yr^{-1})	Inner Mongolia	Under construction
13	Coal-to-oil (IDL)	Yi'tai Yi'li	1.00 (Mt yr^{-1})	Xinjiang	Under construction
14	Coal-to-natural gas	Da'tang Ke'qi Phase I	1.33 (Gm^3 yr^{-1})	Inner Mongolia	In operation
15	Coal-to-natural gas	Qing'hua Yi'li Phase I	1.375 (Gm^3 yr^{-1})	Xinjiang	In operation
16	Coal-to-natural gas	Hui'neng E'er'duo'si Phase I	0.4 (Gm^3 yr^{-1})	Inner Mongolia	In operation
17	Coal-to-natural gas	Zhe'neng Xin'tian Yi'li	2.0 (Gm^3 yr^{-1})	Xinjiang	In operation
18	Coal-to-natural gas	Da'tang Ke'qi Phase II	2.67 (Gm^3 yr^{-1})	Inner Mongolia	Under construction
19	Coal-to-natural gas	Qing'hua Yi'li Phase II	2.625 (Gm^3 yr^{-1})	Xinjiang	Under construction
20	Coal-to-natural gas	Hui'neng E'er'duo'si Phase II	1.2 (Gm^3 yr^{-1})	Inner Mongolia	Under construction
21	Coal-to-natural gas	Da'tang'guo'ji Fu'xin	4.0 (Gm^3 yr^{-1})	Liaoning	Under construction
22	Coal-to-natural gas	Bei'kong E'er'duo'si	4.0 (Gm^3 yr^{-1})	Inner Mongolia	In planning
23	Coal-to-natural gas	Su'xin'neng'yuan He'feng	4.0 (Gm^3 yr^{-1})	Xinjiang	In planning

24	Coal-to-olefin	Shen’hua Bao’tou Phase I	0.6 (Mt yr^{-1})	Inner Mongolia	In operation
25	Coal-to-olefin	Shen’hua Ning’Mei Phase I	0.5 (Mt yr^{-1})	Ningxia	In operation
26	Coal-to-olefin	Shen’hua Ning’Mei Phase II	0.5 (Mt yr^{-1})	Ningxia	In operation
27	Coal-to-olefin	Da’tang Duo’lun	0.46 (Mt yr^{-1})	Inner Mongolia	In operation
28	Coal-to-olefin	Yan’chang Zhong’mei Yu’lin Phase I	0.6 (Mt yr^{-1})	Shaanxi	In operation
29	Coal-to-olefin	Zhong’mei Yu’lin Neng’hua	0.6 (Mt yr^{-1})	Shaanxi	In operation
30	Coal-to-olefin	Bao’feng Phase I	0.6 (Mt yr^{-1})	Ningxia	In operation
31	Coal-to-olefin	Pu’cheng	0.68 (Mt yr^{-1})	Shaanxi	In operation
32	Coal-to-olefin	Shen’hua Gan’quan’pu	0.68 (Mt yr^{-1})	Xinjiang	In operation
33	Coal-to-olefin	Sinopec E’er’duo’si	1.37 (Mt yr^{-1})	Inner Mongolia	In operation
34	Coal-to-olefin	Yan’hu Phase I	0.33 (Mt yr^{-1})	Qinghai	In operation
35	Coal-to-olefin	Yan’chang Yan’an’neng’yuan	0.6 (Mt yr^{-1})	Shaanxi	In operation
36	Coal-to-olefin	Zhong’an’lian’he	0.7 (Mt yr^{-1})	Anhui	In operation
37	Coal-to-olefin	Bao’feng Phase II	0.6 (Mt yr^{-1})	Ningxia	In operation
38	Coal-to-olefin	Yan’chang Zhong’Mei Yu’lin Phase II	0.6 (Mt yr^{-1})	Shaanxi	Under construction
39	Coal-to-olefin	Bao’feng Phase III	0.5 (Mt yr^{-1})	Ningxia	Under construction
40	Coal-to-olefin	Yan’hu Phase II	0.67 (Mt yr^{-1})	Qinghai	In planning
41	Coal-to-ethylene glycol	Tong’liao Jin’mei	0.3 (Mt yr^{-1})	Inner Mongolia	In operation
42	Coal-to-ethylene glycol	He’nan’mei’hua Xin’xiang	0.2 (Mt yr^{-1})	Henan	In operation
43	Coal-to-ethylene glycol	He’nan’mei’hua Pu’yang	0.2 (Mt yr^{-1})	Henan	In operation
44	Coal-to-ethylene glycol	He’nan’mei’hua An’yang	0.2 (Mt yr^{-1})	Henan	In operation
45	Coal-to-ethylene glycol	He’nan’mei’hua Yongcheng	0.2 (Mt yr^{-1})	Henan	In operation
46	Coal-to-ethylene glycol	He’nan’mei’hua Luo’yang	0.2 (Mt yr^{-1})	Henan	In operation
47	Coal-to-ethylene glycol	Hua’lu’heng’sheng Phase I	0.05 (Mt yr^{-1})	Shandong	In operation
48	Coal-to-ethylene glycol	Hua’lu’heng’sheng Phase II	0.5 (Mt yr^{-1})	Shandong	In operation
49	Coal-to-ethylene glycol	Tian’ye Phase I	0.05 (Mt yr^{-1})	Xinjiang	In operation

50	Coal-to-ethylene glycol	Tian'ye Phase II	0.2 (Mt yr^{-1})	Xinjiang	In operation
51	Coal-to-ethylene glycol	Sinopec Hu'hua	0.2 (Mt yr^{-1})	Hubei	In operation
52	Coal-to-ethylene glycol	He'bi Bao'ma Phase I	0.05 (Mt yr^{-1})	Henan	In operation
53	Coal-to-ethylene glycol	Huai'hua	0.1 (Mt yr^{-1})	Anhui	In operation
54	Coal-to-ethylene glycol	E'er'duo'si Xin'hang'neng'yuan	0.3 (Mt yr^{-1})	Inner Mongolia	In operation
55	Coal-to-ethylene glycol	Yang'mei Shen'zhou	0.22 (Mt yr^{-1})	Hebei	In operation
56	Coal-to-ethylene glycol	Yang'mei Shou'yang	0.2 (Mt yr^{-1})	Shanxi	In operation
57	Coal-to-ethylene glycol	Yang'mei Ping'ding	0.2 (Mt yr^{-1})	Shanxi	In operation
58	Coal-to-ethylene glycol	Li'hua Yi'li'jin	0.2 (Mt yr^{-1})	Shandong	In operation
59	Coal-to-ethylene glycol	Qian'xi	0.3 (Mt yr^{-1})	Guizhou	In operation
60	Coal-to-ethylene glycol	Tian'zhi'chen'ye	0.1 (Mt yr^{-1})	Xinjiang	In operation
61	Coal-to-ethylene glycol	Zhong'yan Hong'si'fang	0.3 (Mt yr^{-1})	Anhui	In operation
62	Coal-to-ethylene glycol	Yi'gao San'wei	0.2 (Mt yr^{-1})	Inner Mongolia	In operation
63	Coal-to-ethylene glycol	Rong'xin'hua'gong	0.4 (Mt yr^{-1})	Inner Mongolia	In operation
64	Coal-to-ethylene glycol	Tian'ye syngas	0.6 (Mt yr^{-1})	Xinjiang	In operation
65	Coal-to-ethylene glycol	Mei'hua Bin'chang	0.3 (Mt yr^{-1})	Shaanxi	Under construction
66	Coal-to-ethylene glycol	Jiu'tai E'er'duo'si	1.0 (Mt yr^{-1})	Inner Mongolia	Under construction
67	Coal-to-ethylene glycol	Yu'lin Hua'gong'xin'cai'liao	1.8 (Mt yr^{-1})	Shaanxi	Under construction

Note: DL = direct liquefaction; and IDL = indirect liquefaction.

Supplementary Table 4 2030 projections for national production of traditional coal chemicals in China

Coal chemical	2030 projection (Mt)	Main downstream demand sectors	Reference
Coal-to-coke	395	Steelmaking	Ref[11]
Coal-to-ammonia	45	Nitrogen fertilizer, urea for vehicles, power plant flue gas desulfurization	Ref[11]
Coal-to-methanol	41.25	Alcohol-ether fuel, olefin production	Ref[11]
Coal-to-calcium carbide	27.58	Polyvinyl chloride, butanediol, calcium cyanamide	Same as 2020

Note: We refer to the 2030 projections for coal-to-coke/ammonia/methanol based on a recent study conducted by a national environmental policy agency at Ministry of Ecology and Environment of China[11]. We set the 2030 projection for coal-to-calcium carbide equal to that in 2020 as a relatively stable demand is expected in the near future[12].

Supplementary Table 5 2030 projections for national production of modern coal chemicals in China

Coal chemical	2030 capacity (Mt yr^{-1})	2030 production (Mt)
Coal-to-oil (direct liquefaction)	2.58	2.32
Coal-to-oil (indirect liquefaction)	11.4	10.2
Coal-to-natural gas	2.51	16.3
Coal-to-olefin	12.5	11.2
Coal-to-ethylene glycol	8.57	7.71

Note: We assume individual projects currently under construction or being planned (see Supplementary Table 3) will be operational in 2030. We use a 90% capacity factor[10] for estimation of 2030 production quantities.

Supplementary Table 6 2030 projections for provincial coal chemical production in China

Region	Coal-to-coke in 2030 (Mt)	Coal-to-calcium carbide in 2030 (Mt)	Coal-to-ammonia in 2030 (Mt)	Coal-to-methanol in 2030 (Mt)	Coal-to-oil (direct liquefaction) in 2030 (Mt)	Coal-to-oil (indirect liquefaction) in 2030 (Mt)	Coal-to-natural gas in 2030 (Mt)	Coal-to-olefin in 2030 (Mt)	Coal-to-ethylene glycol in 2030 (Mt)
Beijing	0.00	0.00	0.00	0.00	0.00	0.00	0.00	0.00	0.00
Tianjin	1.47	0.00	0.15	0.25	0.00	0.00	0.00	0.00	0.00
Hebei	40.45	0.00	2.11	1.04	0.00	0.00	0.00	0.00	0.20
Shanxi	87.97	0.35	4.09	2.67	0.00	1.04	0.00	0.00	0.36
Inner Mongolia	35.40	10.18	1.00	7.18	2.32	3.19	6.22	3.27	1.98
Liaoning	19.26	0.06	0.71	0.14	0.00	0.00	2.59	0.00	0.00
Jilin	3.09	0.00	0.37	0.01	0.00	0.00	0.00	0.00	0.00
Heilongjiang	8.91	0.02	0.50	0.42	0.00	0.00	0.00	0.00	0.00
Shanghai	4.53	0.00	0.00	0.95	0.00	0.00	0.00	0.00	0.00
Jiangsu	11.01	0.00	2.67	0.81	0.00	0.00	0.00	0.00	0.00
Zhejiang	1.79	0.00	0.59	0.15	0.00	0.00	0.00	0.00	0.00
Anhui	10.30	0.03	2.72	0.73	0.00	0.00	0.00	0.63	0.36
Fujian	1.87	0.00	0.67	0.30	0.00	0.00	0.00	0.00	0.00
Jiangxi	5.77	0.27	0.07	0.02	0.00	0.00	0.00	0.00	0.00
Shandong	26.51	0.00	5.82	6.29	0.00	0.00	0.00	0.00	0.68
Henan	15.49	0.59	5.06	3.71	0.00	0.00	0.00	0.00	0.95
Hubei	6.72	0.29	3.85	0.44	0.00	0.00	0.00	0.00	0.18
Hunan	5.06	0.10	0.82	0.05	0.00	0.00	0.00	0.00	0.00
Guangdong	5.00	0.00	0.01	0.00	0.00	0.00	0.00	0.00	0.00
Guangxi	6.81	0.00	0.80	0.15	0.00	0.00	0.00	0.00	0.00

Hainan	0.00	0.00	0.60	1.48	0.00	0.00	0.00	0.00	0.00
Chongqing	2.34	0.18	1.65	1.99	0.00	0.00	0.00	0.00	0.00
Sichuan	9.01	1.03	3.04	0.33	0.00	0.00	0.00	0.00	0.00
Guizhou	3.59	0.00	1.86	0.43	0.00	0.00	0.00	0.00	0.27
Yunnan	9.17	0.60	1.85	0.51	0.00	0.00	0.00	0.00	0.00
Tibet	0.00	0.00	0.00	0.00	0.00	0.00	0.00	0.00	0.00
Shaanxi	41.05	2.96	1.49	4.41	0.00	1.49	0.00	2.77	1.89
Gansu	4.33	0.85	0.41	0.35	0.00	0.00	0.00	0.00	0.00
Qinghai	1.53	0.06	0.01	0.15	0.00	0.00	0.00	1.53	0.00
Ningxia	7.72	3.99	0.49	5.17	0.00	3.60	0.00	2.43	0.00
Xinjiang	18.84	6.02	1.61	1.14	0.00	0.90	7.45	0.61	0.86
National	395.00	27.58	45.00	41.25	2.32	10.22	16.26	11.24	7.71

Note: We allocate the 2030 national traditional coal chemical production (Supplementary Table 4) to provincial regions using the same distribution patterns of provincial traditional coal chemical production as in 2020 due to stable production distribution in recent years[4]. We allocate the 2030 national modern coal chemical production (Supplementary Table 5) to provincial regions using the 2030 provincial capacity distribution of modern coal chemical projects (Supplementary Table 3). Provincial capacities of modern coal chemical projects are derived assuming individual projects currently under construction or being planned will be operational in 2030.

Supplementary Table 7 GHG emission factors of coal chemicals in China for 2020 estimates

Coal chemical	GHG emission factor (tCO_2eq/t)			
	Chemical process	Onsite fuel combustion	Upstream process	Total
Coal-to-oil (direct liquefaction)	2.93	1.09	1.22	5.24 (4.23-6.23)
Coal-to-oil (indirect liquefaction)	4.93	2.08	1.89	8.90 (8.06-9.77)
Coal-to-natural gas	4.87	1.51	1.24	7.61 (7.08-8.15)
Coal-to-olefin	6.58	2.38	3.24	12.2 (11.7-12.7)
Coal-to-ethylene glycol	4.19	5.94	1.67	11.8 (10.8-12.8)
Coal-to-coke	0.12	0.033	0.46	0.61 (0.59-0.62)
Coal-to-calcium carbide	1.36	1.81	2.39	5.56 (5.28-5.83)
Coal-to-ammonia	3.14	0.78	0.94	4.87 (4.66-5.09)
Coal-to-methanol	2.55	1.56	0.70	4.81 (4.54-5.06)

Note: GHG emission factors of chemical processes and onsite fuel combustion are cited from Zhang et al[4] for coal chemicals expect calcium carbide and Liu et al[13] for calcium carbide. Upstream processes include those for grid electricity, outsourced heat, and coal mining and processing from a life-cycle perspective. We estimate demands of coal, grid electricity, and outsourced heat for coal chemical production based on literature and reports (Supplementary Table 8), and then use life-cycle GHG emissions factors[14] of various electricity generation technologies (Supplementary Table 9) with a 2020 grid electricity mix (Supplementary Table 10) to derive the life-cycle GHG emissions factor of 2020 grid electricity in China (Supplementary Table 9). In addition, we obtain the life-cycle GHG emission factors of coal mining and processing and outsourced heat supply from a localized life-cycle database for China[14,15] (Supplementary Table 9). Thus, we derive the GHG emission factors of upstream processes for coal chemicals in this table. Total GHG emission factors are the sum of those for chemical processes, onsite fuel combustion, and upstream processes. Low and high estimates of total GHG emission factors of coal chemicals except calcium carbide are derived by uncertainty ranges of carbon emission factors provided in literature[4]. Low and high estimates of total GHG emission factor of coal-to-calcium carbide are determined by 100±5% of the central estimate, respectively.

Supplementary Table 8 Demands for coal, grid electricity, and outsourced heat in coal chemical production of China

Coal chemical	Demand per tonne of coal chemical product			
	Fuel coal (t/t)	Grid electricity (MWh/t)	Outsourced heat (GJ/t)	Feedstock coal (t/t)
Coal-to-oil (direct liquefaction)	0.55	0	0	3.9[16]
Coal-to-oil (indirect liquefaction)	1.04	1.10	0	3.7[16]
Coal-to-natural gas	0.76	0.25	0.54	3.1[16]
Coal-to-olefin	1.20	2.69	0	5.0[17]
Coal-to-ethylene glycol	2.98	0.62	0	2.7[18]
Coal-to-coke	0.017	0.057	0.25	1.4[19]
Coal-to-calcium carbide	0.91	3.40	0	0.81[20]
Coal-to-ammonia	0.39	0.85	0	1.3[21]
Coal-to-methanol	0.78	0.28	0	1.4[22]

Note:

Fuel coal demands are derived using the CO_2 emission factors of onsite fuel combustion per tonne of coal chemicals (reported in literature[4,13]) dividing the CO_2 emission factor of coal combustion (1.981 tCO_2/t, reported in the World Resources Institute GHG accounting protocol[23]).

Grid electricity demands are derived using the CO_2 emission factor of grid electricity per tonne of coal chemicals (reported in literature for around 2015[4,13]) dividing the CO_2 emission factor of 2015 grid electricity (0.6101 tCO_2/MWh, released by Ministry of Ecology and Environment of China[24]).

Outsourced heat demands are derived using the CO_2 emission factors of outsourced heat per tonne of coal chemicals (reported in literature[4]) dividing the CO_2 emission factor of heat supply (0.11 tCO_2/GJ, suggested in China's GHG accounting guidelines for chemical enterprises[25]).

Data sources for feedstock coal demands per tonne of coal chemicals are noted in the table.

Supplementary Table 9 GHG emission factors for fuels, electricity, and heat

GHG emission factor		Value	Metric	Source
Onsite	Coal combustion	1.99	$kgCO_2eq/kg$	Ref [23]
	Natural gas combustion	2.16	$kgCO_2eq/m^3$	Ref [23]
Life-cycle	Coal production for fuel	0.180	$kgCO_2eq/kg$	Ref [14]
	Coal production for feedstock	0.288	$kgCO_2eq/kg$	Ref [14]
	Natural gas production for fuel	0.279	$kgCO_2eq/m^3$	Ref [14]
	Outsourced heat supply	0.126	$kgCO_2eq/MJ$	Ref [14]
	Solar electricity	0.0362	tCO_2eq/MWh	Ref [26]
	Wind electricity (onshore)	0.0199	tCO_2eq/MWh	Ref [27]
	2020 grid electricity	0.586	tCO_2eq/MWh	*
	2030 moderate-renewables grid electricity	0.572	tCO_2eq/MWh	*
	2030 high-renewables grid electricity	0.441	tCO_2eq/MWh	*

*Note: We estimate the life-cycle GHG emission factors of 2020/2030 grid electricity using grid electricity mixes (Supplementary Table 10). We use a national average thermal power generation efficiency of 40.2%[28] for fuel-to-electricity. Thus, life-cycle GHG emission factor of grid electricity = share of coal-based electricity ÷ 40.2% × (onsite GHG emission factor of coal combustion + life-cycle GHG emission factor of coal production for fuel) + share of natural gas-based electricity ÷ 40.2% × (onsite GHG emission factor of natural gas combustion + life-cycle GHG emission factor of natural gas production for fuel) + share of solar electricity × life-cycle GHG emission factor of solar electricity + share of wind electricity × life-cycle GHG emission factor of wind electricity.

Supplementary Table 10 Grid electricity mixes for 2020 and 2030

	Coal	Hydro	Nuclear	Wind	Solar	Gas	Other
2020[29]	0.61	0.18	0.05	0.06	0.03	0.03	0.04
2030 Moderate-renewables[30]	0.57	0.14	0.08	0.07	0.05	0.07	0.02
2030 High-renewables[31]	0.43	0.14	0.07	0.16	0.09	0.06	0.05

Note: We use the 2020 grid electricity mix for estimation of 2020 coal chemical GHG emissions. We use the 2030 Moderate-renewables grid electricity mix for estimation of GHG emissions in the 2030 baseline scenario and 2030 Moderate-renewables Grid Electricity scenario, and use the 2030 High-renewables grid electricity mix for estimation of GHG emissions in the 2030 High-renewables Grid Electricity scenario. Total electricity generation in China was ~7600 TWh in 2020[29] and is projected to be 9400-11800 TWh in 2030[30,31].

Supplementary Table 11 2030 baseline GHG emission factors of coal chemicals in China

Coal chemical	GHG emission factor (tCO_2eq/t)			
	Chemical process	Onsite fuel combustion	Upstream process	Total
Coal-to-oil (direct liquefaction)	2.93	1.09	1.22	5.24 (4.23-6.23)
Coal-to-oil (indirect liquefaction)	4.93	2.08	1.87	8.88 (8.04-9.75)
Coal-to-natural gas	4.87	1.51	1.23	7.61 (7.08-8.15)
Coal-to-olefin	6.58	2.38	3.20	12.2 (11.7-12.7)
Coal-to-ethylene glycol	4.19	5.94	1.66	11.8 (10.8-12.8)
Coal-to-coke	0.12	0.033	0.46	0.61 (0.59-0.62)
Coal-to-calcium carbide	1.36	1.81	2.34	5.51 (5.23-5.79)
Coal-to-ammonia	3.14	0.78	0.93	4.86 (4.65-5.08)
Coal-to-methanol	2.55	1.56	0.70	4.81 (4.54-5.06)

Note: Upstream processes include those for grid electricity, outsourced heat, and coal mining and processing from a life-cycle perspective. In the 2030 baseline scenario, we assume the GHG emission factors of chemical processes, onsite fuel combustion, coal mining and processing, and outsourced heat to be the same as those in 2020 (Supplementary Table 7). We use a 2030 moderate grid electricity mix (Supplementary Table 10) to calculate the life-cycle GHG emission factor of 2030 moderate grid electricity (Supplementary Table 9) for the 2030 baseline scenario. Thus, we derive the GHG emission factors of upstream processes for coal chemicals in the table. Total GHG emission factors are the sum of those for chemical processes, onsite fuel combustion, and upstream processes. Low and high estimates of total GHG emission factors of coal chemicals except calcium carbide are derived by uncertainty ranges of carbon emission factors provided in literature[4]. Low and high estimates of total GHG emission factor of coal-to-calcium carbide are determined by 100±5% of the central estimate, respectively.

Supplementary Table 12 Chemical reaction equations of coal chemical production

Coal chemical product	Formula	Chemical reaction	H_2/CO ratio of syngas for further synthesis
Coal-to-oil (direct liquefaction)	C_nH_{2n+2}	$nC + (n+1)H_2 \rightarrow C_nH_{2n+2}$, where H_2 is mainly from coal gasification and water-gas shift	Only H_2 needed
Coal-to-oil (indirect liquefaction)	C_nH_{2n+2}	Coal gasification, water-gas shift, $nCO + (2n+1)H_2 \rightarrow C_nH_{2n+2} + nH_2O$	(2n+1)/n
Coal-to-natural gas	CH_4	Coal gasification, water-gas shift, $CO + 3H_2 \rightarrow CH_4 + H_2O$	3
Coal-to-olefin	$(CH_2)_n$	Coal gasification, water-gas shift, $CO + 2H_2 \rightarrow CH_3OH$, $2CH_3OH \rightarrow C_2H_4 + 2H_2O$ / $3CH_3OH \rightarrow C_3H_6 + 3H_2O$	2
Coal-to-ethylene glycol	$C_2H_4(OH)_2$	Coal gasification, water-gas shift, $2CO + 4H_2 + 1/2O_2 \rightarrow C_2H_4(OH)_2 + H_2O$	2
Coal-to-coke	C	$C \rightarrow C$	No H_2 and CO needed
Coal-to-calcium carbide	CaC_2	$CaCO_3 \rightarrow CaO + CO_2$, $CaO + 3C \rightarrow CaC_2 + CO$	No H_2 and CO needed
Coal-to-ammonia	NH_3	Coal gasification, water-gas shift, $N_2 + 3H_2 \rightarrow 2NH_3$	Only H_2 needed
Coal-to-methanol	CH_3OH	Coal gasification, water-gas shift, $CO + 2H_2 \rightarrow CH_3OH$	2

Note: coal gasification, $2C + O_2 \rightarrow 2CO$ / $C + H_2O \rightarrow CO + H_2$ / $C + O_2 \rightarrow CO_2$; the water-gas shift, $CO + H_2O \rightarrow CO_2 + H_2$; n = 10-20[32], and we assume n = 15 in our analysis.

Supplementary Table 13 CO_2 emission factors and H_2/O_2 demands of coal chemicals in China

Coal chemical product	CO_2 emission factor (tCO_2/t)			H_2 and O_2 demand per tonne of coal chemicals (t/t)			
	Whole chemical process [a]	Water-gas shift [b]	Reductions in whole chemical process using electrolytic H_2 [c]	Water-gas shift-based H_2 demand [d]	Air separation-based O_2 demand [e]	Electrolytic H_2 demand [f]	Electrolytic O_2 demand [g]
Coal-to-oil (direct liquefaction)	2.93	2.86	2.93	0.130	0.61	0.190	0
Coal-to-oil (indirect liquefaction)	4.93	4.71	4.82	0.214	1.92	0.313	0.92
Coal-to-natural gas	4.87	4.69	4.80	0.213	1.58	0.311	0.58
Coal-to-olefin	6.58	6.28	6.43	0.285	2.62	0.417	1.27
Coal-to-ethylene glycol	4.19	4.00	4.10	0.182	2.36	0.266	1.50
Coal-to-coke	0.12	0	0	0	0	0	0
Coal-to-calcium carbide	1.36	0	0	0	0	0	0
Coal-to-ammonia	3.14	3.06	3.14	0.139	0.65	0.204	0
Coal-to-methanol	2.55	2.43	2.49	0.111	1.01	0.162	0.49

Note:

(a) *CO_2 emission factors of the whole chemical process* for each coal chemical are cited from literature[4,13]. We use a typical mixture for the syngas from coal gasification, which includes CO (65%), H_2 (30%), CO_2 (1.6%), N_2+Ar (3.1%), and other gases (0.3%)[33,34]. We assume the volumes of CO, H_2, and CO_2 in the syngas to be 65, 30, and 1.6 in calculations below.

(b) CO_2 emissions of coal chemical production mostly result from coal gasification and the water-gas shift reaction. We assume the volume of CO that is converted to H_2 *via* the water-gas shift to be x, where x is in the range of 0-65. Thus, *CO_2 emission factors of the water-gas shift* = *CO_2 emission factors of the whole chemical process* × $x/(x+1.6)$. We obtain the H_2/CO ratios (r) for further syntheses based on chemical reaction equations listed in Supplementary Table 12, and the initial H_2/CO ratio = 30/65. Thus, we derive that $(30+x)/(65-x) = r$, and then $x = (65×r-30)/(r+1)$.

(c) As feedstock coal is used with O_2 and water steam to generate CO and H_2 for further syntheses, using electrolytic H_2 can reduce the portion of coal

that is used for only generating H_2. This portion of coal is first gasified to generate CO and H_2 where emitting a small quantity of CO_2 emissions, and then CO with water steam is converted to H_2 and CO_2 *via* the water-gas shift. Thus, reduced CO_2 emissions include those from both gasification and the water-gas shift of the portion of coal. Accordingly, we derive that *Reductions in CO_2 emission factors of the whole chemical process using electrolytic H_2* = *CO_2 emission factors of the water-gas shift* × (65+1.6)/65.

(d) According to the chemical reaction equation ($CO + H_2O \rightarrow CO_2 + H_2$) listed in Supplementary Table 12, *Water-gas shift-based H_2 demand* = *CO_2 emission factors of the water-gas shift* ÷ 44 (molecular weight of CO_2) × 2 (molecular weight of H_2).

(e) O_2 is used for coal gasification where generating both CO and CO_2. According to the chemical reaction equations ($2C + O_2 \rightarrow 2CO$, $C + H_2O \rightarrow CO + H_2$, $C + O_2 \rightarrow CO_2$) listed in Supplementary Table 12, the O element of CO_2 is from O_2 and the O element of CO is from both O_2 and H_2O. For coal chemicals except ethylene glycol, *Air separation-based O_2 demand* = *CO_2 emission factors of the water-gas shift* ÷ 44 (molecular weight of CO_2) ÷ x × ((65-30) × 16 (relative atomic mass of O) + 1.6 × 16 (relative atomic mass of O) × 2). For ethylene glycol, O_2 is also used in further synthesis ($2CO + 4H_2 + 1/2O_2 \rightarrow C_2H_4(OH)_2 + H_2O$), thus *Air separation-based O_2 demand* = *CO_2 emission factors of the water-gas shift* ÷ 44 (molecular weight of CO_2) ÷ x × ((65-30) × 16 (relative atomic mass of O) + 1.6 × 16 (relative atomic mass of O) × 2 + (30+x)/4 × 16 (relative atomic mass of O)).

(f) Electrolytic H_2 is used to replace coal-based H_2 that is produced from both coal gasification and the water-gas shift reaction. Removal of the water-gas shift results in reductions in H_2 production from coal gasification due to coal input decrease. Based on above-mentioned syngas mixture from coal gasification, *Electrolytic H_2 demand* = *Water-gas shift-based H_2 demand* × (65+30)/65.

(g) Electrolytic O_2 is used to replace air separation-based O_2 for coal gasification. We obtain the H_2/CO ratios (r) for further syntheses based on chemical reaction equations listed in Supplementary Table 12. We assume the volume of *electrolytic H_2 demand* to be y. Thus, we derive that (30+y)/65 = r, and then $y = 65 \times r - 30$. For coal chemicals except ethylene glycol, *Electrolytic O_2 demand* = *Electrolytic H_2 demand* ÷ 2 (molecular weight of H_2) ÷ y × ((65-30) × 16 (relative atomic mass of O) + 1.6 × 16 (relative atomic mass of O) × 2). For ethylene glycol, *Electrolytic O_2 demand* = *Electrolytic H_2 demand* ÷ 2 (molecular weight of H_2) ÷ y × ((65-30) × 16 (relative atomic mass of O) + 1.6 × 16 (relative atomic mass of O) × 2 + (30+y)/4 × 16 (relative atomic mass of O)).

Supplementary Table 14 GHG emission changes of four alternative scenarios relative to the baseline scenario by provincial region and by individual process

Provincial region	GHG emission changes ($MtCO_2eq$)														
	Coal-based H_2	Air separation -based O_2	Coal gasification	Coal production	MG for grid electricity	Electrolyzer manufacture	Battery manufacture	Leakage to atmosphere	MG for water electrolysis	HG for water electrolysis	HG for grid electricity	SE for water electrolysis	SE to replace grid electricity	WE for water electrolysis	WE to replace grid electricity
	a	***b***	***c***	***d***	***e***	***f***	***g***	***h***	***i***	***j***	***k***	***l***	***m***	***n***	***o***
Beijing	0.00	0.00	0.00	0.00	-1.01	0.00	0.00	0.00	8.47	6.54	0.78	0.21	0.03	0.00	0.00
Tianjin	-1.09	-0.08	-0.07	0.00	-1.72	0.01	0.01	0.02	14.39	11.11	1.33	0.45	0.05	0.07	0.005
Hebei	-10.01	-0.69	-0.61	-1.00	-6.58	0.06	0.13	0.15	54.99	42.44	5.08	3.39	0.40	0.63	0.08
Shanxi	-26.00	-1.96	-1.59	-21.65	-7.05	0.15	0.34	0.40	58.89	45.45	5.44	2.37	0.28	2.14	0.26
Inner Mongolia	-102.21	-9.17	-6.25	-20.57	-11.25	0.58	1.36	1.58	94.02	72.56	8.68	0.29	0.03	9.20	1.31
Liaoning	-15.01	-1.12	-0.92	-0.63	-3.50	0.09	0.20	0.23	29.26	22.58	2.70	0.16	0.02	1.10	0.05
Jilin	-1.18	-0.06	-0.07	-0.21	-1.74	0.01	0.02	0.02	14.57	11.24	1.35	0.00	0.00	0.07	0.01
Heilongjiang	-2.61	-0.18	-0.16	-1.12	-2.14	0.01	0.03	0.04	17.90	13.82	1.65	0.00	0.00	0.16	0.02
Shanghai	-2.38	-0.23	-0.15	0.00	-1.94	0.01	0.03	0.04	16.18	12.48	1.49	1.21	0.14	0.13	0.01
Jiangsu	-10.40	-0.61	-0.64	-0.21	-10.30	0.06	0.14	0.16	86.11	66.45	7.95	24.49	2.87	1.01	0.08
Zhejiang	-2.22	-0.13	-0.14	0.00	-5.68	0.01	0.03	0.03	47.44	36.61	4.38	7.70	0.90	0.14	0.01
Anhui	-15.89	-1.20	-0.97	-2.22	-5.94	0.09	0.21	0.25	49.63	38.31	4.58	6.23	0.73	1.57	0.13
Fujian	-2.85	-0.18	-0.17	-0.13	-3.64	0.02	0.04	0.04	30.40	23.46	2.81	0.58	0.07	0.19	0.01
Jiangxi	-0.26	-0.02	-0.02	-0.06	-2.71	0.00	0.00	0.00	22.67	17.50	2.09	4.99	0.59	0.02	0.03
Shandong	-36.71	-2.81	-2.24	-2.20	-11.95	0.21	0.49	0.57	99.85	77.06	9.22	0.15	0.02	2.43	0.15
Henan	-29.01	-2.22	-1.77	-2.14	-5.84	0.17	0.39	0.45	48.84	37.70	4.51	1.43	0.17	3.80	0.31
Hubei	-13.91	-0.81	-0.85	-0.01	-2.84	0.08	0.18	0.22	23.74	18.32	2.19	0.56	0.07	1.10	0.10
Hunan	-2.69	-0.14	-0.16	-0.21	-1.98	0.02	0.04	0.04	16.54	12.77	1.53	0.00	0.00	0.23	0.03
Guangdong	-0.02	0.00	0.00	0.00	-8.37	0.00	0.00	0.00	69.97	54.00	6.46	1.98	0.23	0.00	0.01
Guangxi	-2.89	-0.16	-0.18	-0.08	-2.47	0.02	0.04	0.04	20.68	15.96	1.91	0.01	0.00	0.29	0.03
Hainan	-5.58	-0.45	-0.34	0.00	-0.50	0.03	0.07	0.09	4.14	3.19	0.38	0.07	0.01	0.36	0.02
Chongqing	-10.12	-0.74	-0.62	-0.19	-1.26	0.06	0.13	0.16	10.52	8.12	0.97	0.04	0.00	0.85	0.06
Sichuan	-10.37	-0.55	-0.63	-0.45	-1.23	0.06	0.14	0.16	10.25	7.91	0.95	2.43	0.29	0.63	0.11
Guizhou	-8.02	-0.55	-0.49	-2.42	-3.10	0.05	0.11	0.12	25.87	19.97	2.39	0.26	0.03	0.62	0.04

Yunnan	-7.07	-0.41	-0.43	-1.11	-0.96	0.04	0.09	0.11	8.05	6.21	0.74	0.07	0.01	0.49	0.08
Tibet	0.00	0.00	0.00	0.00	-0.01	0.00	0.00	0.00	0.08	0.06	0.01	0.00	0.00	0.00	0.00
Shaanxi	-48.42	-4.77	-2.96	-13.64	-4.73	0.28	0.64	0.75	39.58	30.54	3.65	5.82	0.68	3.11	0.43
Gansu	-2.16	-0.15	-0.13	-0.77	-2.04	0.01	0.03	0.03	17.01	13.13	1.57	0.00	0.00	0.18	0.08
Qinghai	-10.25	-0.99	-0.63	-0.22	-0.24	0.06	0.14	0.16	2.03	1.57	0.19	0.32	0.04	0.93	0.11
Ningxia	-47.43	-4.49	-2.90	-1.64	-3.55	0.27	0.63	0.73	29.71	22.93	2.74	0.00	0.00	3.17	0.47
Xinjiang	-55.49	-4.60	-3.39	-5.41	-7.58	0.32	0.74	0.86	63.36	48.90	5.85	0.00	0.00	3.44	0.47

Note: MG = Moderate Grid Electricity; HG = High-renewables Grid Electricity; SE = Onsite Solar Electricity; and WE = Onsite Wind Electricity.

GHG mitigation of the MG scenario = $a + b + c + d + f + h + i$; GHG mitigation of the HG scenario = $a + b + c + d + e + f + h + j + k$; GHG mitigation of the SE scenario = $a + b + c + d + e + f + g + h + l + m$; and GHG mitigation of the WE scenario = $a + b + c + d + e + f + g + h + n + o$. See detailed configurations of the baseline scenario and four alternative scenarios in Table 1 and related descriptions in the main text.

We use the 2030 provincial production of coal chemicals (Supplementary Table 6) and demands per tonne of coal chemicals for electrolytic H_2 and O_2 (Supplementary Table 13) and purchased grid electricity (Supplementary Table 8) to estimate the provincial demands for electrolytic H_2 and O_2 and purchased grid electricity. Then we characterize GHG emission changes by provincial region and by individual process using these provincial demands with GHG emission factors of coal chemical processes (Supplementary Table 13), coal production, moderate/high-renewables grid electricity (Supplementary Table 9), air separation, coal gasification, electrolyzer and battery manufacture, H_2 leakage (Supplementary Table 15), and provincial solar/wind electricity (Supplementary Table 16).

GHG emission changes resulting from variations in coal chemical production are attributed to provincial regions where these changes physically occur. Onsite GHG emission changes occur in local provincial regions where coal chemicals are produced, including changes in chemical processes, onsite fuel combustion for air separation and coal gasification, and H_2 leakage to the atmosphere. We allocate GHG emission changes in coal production, grid electricity, and solar power facility manufacture to provincial regions based on the provincial production levels of coal, thermal power, and solar panels, respectively (see Supplementary Table 17)[35,36]. We attribute wind power-related GHG emissions to local provincial regions where wind turbines are deployed, since building materials and nacelles (mainly consisting of concrete and steel) are responsible for the majority of environmental impacts[37] and such materials and equipment have short cost-effective transport distances. We also assume that electrolyzer and battery manufacturing occurs in local provincial regions. Such an attribution approach for upstream environmental impacts has been applied in related studies[38,39].

Supplementary Table 15 Parameters of coal-based H_2 production, air separation, coal gasification, water electrolysis, and battery storage

Parameter	Metric	Reference
Feedstock coal needed for H_2 production *via* coal gasification and the water-gas shift reaction	8.0 t coal/t H_2	Ref[40,41]
Life-cycle GHG emission factor of coal mining and processing for H_2 production [a]	1.44 t CO_2eq/t H_2	Ref[14,40,41]
Electricity needed for O_2 production *via* air separation	0.280 MWh/t O_2	Ref[33]
Life-cycle GHG emission factor of coal-based electricity generation [b]	0.929 t CO_2eq/MWh	Ref[14,28]
Coal needed for electricity generation to drive air separation for O_2 production [c]	0.120 t coal/t O_2	Ref[28,33]
GHG emission factor of onsite coal combustion for O_2 production [d]	0.238 t CO_2eq/t O_2	Ref[23,28,33]
Life-cycle GHG emission factor of coal mining and processing for O_2 production [e]	0.0215 t CO_2eq/t O_2	Ref[14,28,33]
Life-cycle GHG emission factor of coal-driven air separation-based O_2 production [f]	0.260 t CO_2eq/t O_2	Ref[14,23,28,33]
Electricity needed for coal gasification	0.138 MWh/t coal	Ref[42,43]
Coal needed for electricity generation to drive coal gasification [g]	0.0591 t/t coal	Ref[28,42]
GHG emission factor of onsite coal combustion for coal gasification [h]	0.118 t CO_2eq /t coal	Ref[23,28,42]
Life-cycle GHG emission factor of coal mining and processing for coal gasification [i]	0.0106 t CO_2eq /t coal	Ref[14,28,42]
Life-cycle GHG emission factor of coal gasification [j]	0.128 t CO_2eq /t coal	Ref[14,23,28,42]
Electricity needed for H_2 production *via* water electrolysis [k]	57.9 MWh/t H_2	Ref[44]

Leakage rate of H_2 during production	5.0%	Ref[45,46]
Leakage rate of H_2 to the atmosphere	2.06%	Ref[45,46]
GHG emission factor of H_2 leakage during H_2 production *l*	0.227 t CO_2eq/t H_2	Ref[45-47]
Life-cycle GHG emission factor of electrolyzer manufacture needed for H_2 production *m*	0.083 t CO_2eq/t H_2	Ref[44]
Life-cycle GHG emission factor of battery manufacture needed for H_2 production *n*	0.194 t CO_2eq/t H_2	Ref[44]

Note:

(a) Life-cycle GHG emission factor of coal mining and processing for H_2 production = Feedstock coal needed for H_2 production via coal gasification and the water-gas shift reaction (8.0 t coal/t H_2[40,41]) × Life-cycle GHG emission factor of coal mining and processing (0.180 tCO_2eq/t coal[14]) = 1.44 tCO_2eq/t H_2.

(b) Life-cycle GHG emission factor of coal-based electricity generation = 3.6 GJ/MWh ÷ Average thermal power generation efficiency (40.2%[28]) ÷ 20.9GJ/t coal × (Life-cycle GHG emission factor of coal mining and processing (0.180 tCO_2eq/t coal[14]) + GHG emission factor of coal combustion (1.99 tCO_2eq/t coal[23])) = 0.929 tCO_2eq/MWh, where 1 MWh=3.6 GJ and 1 t coal=20.9 GJ.

(c) Coal needed for electricity generation to drive air separation for O_2 production = Electricity needed for O_2 production *via* air separation (0.280 MWh/tO_2[33]) × 3.6 GJ/MWh ÷ Average thermal power generation efficiency (40.2%[28]) ÷ 20.9GJ/t coal = 0.120 t coal/t O_2, where 1 MWh=3.6 GJ and 1 t coal=20.9 GJ.

(d) GHG emission factor of onsite coal combustion for O_2 production = Coal needed for electricity generation to drive air separation for O_2 production (0.120 t coal/t O_2) × GHG emission factor of coal combustion (1.99 tCO_2eq/t coal[23]) = 0.238 tCO_2eq/t O_2.

(e) Life-cycle GHG emission factor of coal mining and processing for O_2 production = Coal needed for electricity generation to drive air separation for O_2 production (0.120 t coal/t O_2) × Life-cycle GHG emission factor of coal mining and processing (0.180 tCO_2eq/t coal[14]) = 0.0215 tCO_2eq/t O_2.

(f) Life-cycle GHG emission factor of air separation-based O_2 production = GHG emission factor of onsite coal combustion for O_2 production (0.238 tCO_2eq/t O_2) + Life-cycle GHG emission factor of coal mining and processing for O_2 production (0.0215 tCO_2eq/t O_2) = Electricity needed

for O_2 production *via* air separation (0.280 MWh/t O_2) × Life-cycle GHG emission factor of coal-based electricity generation (0.929 t CO_2eq/MWh) = 0.260 tCO_2eq/t O_2.

(g) Coal needed for electricity generation to drive coal gasification = Electricity needed for coal gasification (0.138 MWh/t coal[42,43]) × 3.6 GJ/MWh ÷ Average thermal power generation efficiency (40.2%[28]) ÷ 20.9GJ/t coal = 0.0591 t /t coal, where 1 MWh=3.6 GJ and 1 t coal=20.9 GJ.

(h) GHG emission factor of onsite coal combustion for coal gasification = Coal needed for electricity generation to drive coal gasification (0.0591 t/t coal) × GHG emission factor of coal combustion (1.99 tCO_2eq/t coal[23]) = 0.118 tCO_2eq/t coal.

(i) Life-cycle GHG emission factor of coal mining and processing for coal gasification = Coal needed for electricity generation to drive coal gasification (0.0591 t coal/t coal) × Life-cycle GHG emission factor of coal mining and processing (0.180 tCO_2eq/t coal[14]) = 0.0106 tCO_2eq/t coal.

(j) Life-cycle GHG emission factor of coal gasification = GHG emission factor of onsite coal combustion for coal gasification (0.118 tCO_2eq/t) + Life-cycle GHG emission factor of coal mining and processing for coal gasification (0.0106 tCO_2eq/t) = Electricity needed for coal gasification (0.138 MWh/t[42,43]) × Life-cycle GHG emission factor of coal-based electricity generation (0.929 t CO_2eq/MWh) = 0.128 tCO_2eq/t coal.

(k) Electricity needed for H_2 production via water electrolysis is reported to be 55 (50-60) MWh/t H_2 in literature[44]. We consider the leakage rate of H_2 during H_2 production and thus use 55÷(100%-5.0%) = 57.9 MWh/t H_2 in our analysis.

(l) We include H_2 leakage in our analysis which adds to global warming because H_2 is an indirect GHG and extends the lifetime of CH_4 in the atmosphere. We use 100-year global warming potential of 11 for H_2[47] and quantify the GHG emission factor of H_2 leakage during H_2 production to be 2.06%[45,46]×11=0.227 t CO_2eq/t H_2.

(m) A typical water electrolysis project of 10 MW with H_2 production of 936 tonne per year is reported to be operational in practice[48]. We estimate that 31 Mt per year of electrolytic H_2 is required to replace coal-based H_2 in coal chemical production (= 2030 national production of coal chemicals (Supplementary Tables 4-5) × Electrolytic H_2 demand per tonne of coal chemicals (Supplementary Table 13)). Thus, we estimate that 334 GW (=31 Mt÷936 t×10 MW) of water electrolyzers is needed in the four alternative scenarios.

(n) A typical water electrolysis project is analyzed in literature that generates 36500 tonne H_2 per year with battery storage of 375MW[44]. Thus, we estimate that 321 GW (=31 Mt÷36500 t×375 MW) of battery storage is needed in the SE and WE scenarios.

Supplementary Table 16 Provincial GHG emission factors of solar and wind electricity

Region	Solar capacity factor	Wind capacity factor	GHG emission factor of solar electricity ($kgCO_2eq/MWh$)	GHG emission factor of wind electricity ($kgCO_2eq/MWh$)
Beijing	0.205	0.311	37.47	16.27
Tianjin	0.198	0.291	38.88	17.37
Hebei	0.206	0.301	37.23	16.80
Shanxi	0.217	0.231	35.47	21.93
Inner Mongolia	0.236	0.211	32.59	23.97
Liaoning	0.200	0.259	38.52	19.54
Jilin	0.196	0.330	39.17	15.30
Heilongjiang	0.190	0.319	40.46	15.84
Shanghai	0.185	0.337	41.54	14.98
Jiangsu	0.190	0.195	40.39	25.87
Zhejiang	0.194	0.299	39.61	16.88
Anhui	0.191	0.193	40.30	26.25
Fujian	0.212	0.287	36.19	17.63
Jiangxi	0.193	0.214	39.84	23.66
Shandong	0.196	0.287	39.17	17.63
Henan	0.191	0.145	40.34	34.91
Hubei	0.185	0.240	41.54	21.03
Hunan	0.181	0.225	42.55	22.47
Guangdong	0.211	0.221	36.39	22.90
Guangxi	0.203	0.191	37.95	26.44
Hainan	0.238	0.297	32.35	17.04
Chongqing	0.167	0.225	46.15	22.47
Sichuan	0.212	0.311	36.32	16.27
Guizhou	0.205	0.247	37.53	20.43
Yunnan	0.263	0.271	29.19	18.63
Tibet	0.340	0.127	22.63	39.95
Shaanxi	0.210	0.295	36.62	17.12
Gansu	0.236	0.225	32.61	22.47
Qinghai	0.286	0.208	26.84	24.30
Ningxia	0.235	0.284	32.65	17.80
Xinjiang	0.212	0.306	36.24	16.49

Note:

Solar/wind capacity factor = Annual electricity generation of facility (MWh) ÷ (Facility capacity (MW) × 8760 h/a). We obtain up-to-date provincial capacity factors for solar and wind power from literature[49,50]. According to technical projections, we assume solar and wind capacity factors

in China to increase by 10% during 2020-2030[51,52].

Life-cycle GHG emission factor of solar/wind electricity (kgCO_2eq/MWh) = GHG emissions of facility manufacturing and operations ÷ lifetime electricity generation, where lifetime electricity generation = lifetime years × capacity factor × rated annual electricity generation. This indicates GHG emission factor is proportional to (capacity factor)$^{-1}$. We obtain up-to-date national average of life-cycle GHG emission factors of solar PV (36.2 kgCO_2eq/MWh) and onshore wind electricity (19.9 kgCO_2eq/MWh) from literature[26,27]. Thus, we derive that provincial GHG emission factor = national average GHG emission factor × national average capacity factor ÷ provincial capacity factor.

Supplementary Table 17 Provincial production of coal, thermal power, and solar panels

Provincial region	Thermal electricity generation in 2020 (TWh)[35]	Coal production in 2020 (Mt)[35]	Solar cell production in 2018 (GW)[36]
Beijing	44	0	0.32
Tianjin	74	0	0.66
Hebei	283	50	5.00
Shanxi	303	1079	3.49
Inner Mongolia	484	1026	0.43
Liaoning	151	31	0.24
Jilin	75	10	0.00
Heilongjiang	92	56	0.00
Shanghai	83	0	1.78
Jiangsu	443	10	36.06
Zhejiang	244	0	11.34
Anhui	256	111	9.17
Fujian	157	7	0.86
Jiangxi	117	3	7.34
Shandong	514	109	0.22
Henan	252	106	2.11
Hubei	122	0	0.82
Hunan	85	11	0.00
Guangdong	360	0	2.92
Guangxi	106	4	0.02
Hainan	21	0	0.11
Chongqing	54	9	0.06
Sichuan	53	22	3.58
Guizhou	133	121	0.38
Yunnan	41	55	0.11
Tibet	0	0	0.00
Shaanxi	204	680	8.57
Gansu	88	39	0.00
Qinghai	10	11	0.47
Ningxia	153	82	0.00
Xinjiang	326	270	0.00
Total	5330	3902	96.05

Note: We allocate national GHG emission changes in coal production, grid electricity, and PV facility manufacture to provincial regions based on provincial production levels of coal, thermal power, and solar cells (using the ratios of provincial production to national total).

Supplementary Table 18 National average GHG emission factors of coal chemicals in the Onsite Solar Electricity (SE) and Onsite Wind Electricity (WE) scenarios

Coal chemical	GHG emission factor (tCO_2eq/t product)							
	National average in the SE scenario				National average in the WE scenario			
	Process[a]	Fuel[b]	Upstream[c]	Total	Process[a]	Fuel[b]	Upstream[c]	Total
Coal-to-oil (DL)	0.00	0.77	1.25	2.02	0.00	0.77	1.07	1.84
Coal-to-oil (IDL)	0.11	1.33	1.32	2.75	0.11	1.33	1.00	2.43
Coal-to-natural gas	0.07	0.84	1.14	2.04	0.07	0.84	0.84	1.75
Coal-to-olefin	0.15	1.36	1.80	3.31	0.15	1.36	1.37	2.88
Coal-to-ethylene glycol	0.09	5.12	1.34	6.56	0.09	5.12	1.08	6.30
Coal-to-coke	0.12	0.03	0.42	0.58	0.12	0.03	0.42	0.58
Coal-to-calcium carbide	1.36	1.81	0.52	3.69	1.36	1.81	0.46	3.63
Coal-to-ammonia	0.00	0.44	0.51	0.95	0.00	0.44	0.30	0.74
Coal-to-methanol	0.06	1.16	0.57	1.79	0.06	1.16	0.41	1.63

Note: DL = direct liquefaction; and IDL = indirect liquefaction.

(a) Chemical process-related GHG emission factors in the SE/WE scenario = 2030 baseline CO_2 emission factors of whole chemical process (Supplementary Table 13) – Reductions in CO_2 emission factors of whole chemical process using electrolytic H_2 (Supplementary Table 13).

(b) Onsite fuel combustion-related GHG emission factors in the SE/WE scenario =
2030 baseline GHG emission factors of onsite fuel combustion (Supplementary Table 11)
– Air separation-based O_2 demand per tonne of coal chemicals (Supplementary Table 13) × GHG emission factor of onsite coal combustion for O_2 production (Supplementary Table 15)
– Reductions in coal-based H_2 demand per tonne of coal chemicals (= Electrolytic H_2 demand per tonne in Supplementary Table 13) × Feedstock coal needed for H_2 production (Supplementary Table 15) × GHG emission factor of onsite coal combustion for coal gasification (Supplementary Table 15).

(c) Upstream process-related GHG emission factors in the SE/WE scenario =
(Feedstock coal demand per tonne of coal chemicals (Supplementary Table 8) – Reductions in coal-based H_2 demand per tonne of coal chemicals (= Electrolytic H_2 demand per tonne in Supplementary Table 13) × Feedstock coal needed for H_2 production (Supplementary Table 15)) × GHG emission factor of coal production for feedstock (Supplementary Table 9)
+ (Fuel coal demand per tonne of coal chemicals (Supplementary Table 8) – Air separation-based O_2 demand per tonne of coal chemicals (Supplementary Table 13) × Coal needed for electricity generation to drive air separation for O_2 production (Supplementary Table 15) – Reductions in coal-based H_2 demand per tonne of coal chemicals (= Electrolytic H_2 demand per tonne in

Supplementary Table 13) × Feedstock coal needed for H_2 production (Supplementary Table 15) × Coal needed for electricity generation to drive coal gasification (Supplementary Table 15)) × GHG emission factor of coal production for fuel (Supplementary Table 9)

+ Outsourced heat demand per tonne of coal chemicals (Supplementary Table 8) × GHG emission factor of outsourced heat supply (Supplementary Table 9)

+ Electrolytic H_2 demand per tonne of coal chemicals (Supplementary Table 13) ÷ (100% - H_2 leakage rate during production (Supplementary Table 15)) × (GHG emission factor of electrolyzer manufacture needed for H_2 production (Supplementary Table 15) + GHG emission factor of battery manufacture needed for H_2 production (Supplementary Table 15) + GHG emission factor of H_2 leakage during H_2 production (Supplementary Table 15))

+ (Electrolytic H_2 demand per tonne of coal chemicals (Supplementary Table 13) × Electricity needed for H_2 production *via* water electrolysis (Supplementary Table 15) + Grid electricity demand per tonne of coal chemicals (Supplementary Table 8)) × National average GHG emission factor of solar/wind electricity (Supplementary Table 9).

Supplementary Table 19 Remaining GHG emissions in the Onsite Solar Electricity (SE) and Onsite Wind Electricity (WE) scenarios

Coal chemical	Remaining GHG emissions in SE scenario ($MtCO_2eq$)				Remaining GHG emissions in WE scenario ($MtCO_2eq$)			
	Process[a]	Fuel[b]	Upstream[c]	Total	Process[a]	Fuel[b]	Upstream[c]	Total
Coal-to-oil (DL)	0.00	1.79	2.81	4.60	0.00	1.79	2.59	4.38
Coal-to-oil (IDL)	1.08	13.55	12.98	27.60	1.08	13.55	10.26	24.88
Coal-to-natural gas	1.08	13.61	18.27	32.96	1.08	13.61	13.70	28.39
Coal-to-olefin	1.65	15.33	19.44	36.42	1.65	15.33	15.60	32.58
Coal-to-ethylene glycol	0.72	39.53	10.36	50.61	0.72	39.53	8.56	48.81
Coal-to-coke	47.40	13.09	167.82	228.31	47.40	13.09	167.45	227.94
Coal-to-calcium carbide	37.37	49.98	14.17	101.52	37.37	49.98	12.86	100.21
Coal-to-ammonia	0.00	19.60	24.12	43.72	0.00	19.60	14.79	34.38
Coal-to-methanol	2.35	47.97	23.57	73.89	2.35	47.97	17.41	67.72
Total	91.65	214.44	2.81	599.64	91.65	214.44	2.59	569.30

Note: DL = direct liquefaction; and IDL = indirect liquefaction.

(a) Remaining GHG emissions of coal chemical processes in the SE/WE scenario = Chemical process-related GHG emission factors in the SE/WE scenario (Supplementary Table 18) × 2030 national production of coal chemicals (Supplementary Tables 4-5).

(b) Remaining GHG emissions of onsite fuel combustion in the SE/WE scenario = Onsite fuel combustion-related GHG emission factors in the SE/WE scenario (Supplementary Table 18) × 2030 national production of coal chemicals (Supplementary Tables 4-5).

(c) Remaining GHG emissions of upstream processes in the SE/WE scenario =
2030 national production of coal chemicals (Supplementary Tables 4-5) ×
(Upstream process-related GHG emission factors in the SE/WE scenario (Supplementary Table 18)
– (Electrolytic H_2 demand per tonne of coal chemicals (Supplementary Table 13) × Electricity needed for H_2 production *via* water electrolysis (Supplementary Table 15) + Grid electricity demand per tonne of coal chemicals (Supplementary Table 8)) × National average GHG emission factor of solar/wind electricity (Supplementary Table 9))
+ Σ(2030 provincial production of coal chemicals (Supplementary Table 6) × (Electrolytic H_2 demand per tonne of coal chemicals (Supplementary Table 13) × Electricity needed for H_2 production *via* water electrolysis (Supplementary Table 15) + Grid electricity demand per tonne of coal chemicals (Supplementary Table 8))× Provincial GHG emission factor of solar/wind electricity (Supplementary Table 16)).

Supplementary Table 20 Cost parameters for coal chemical production and water electrolysis (in 2020 CNY)

Indicator	Central estimate of 2030 price	Low to high estimates of 2030 price	Reference
Fuel coal price	670 CNY/t	570-770 CNY/t	Ref[53]
Feedstock coal price	1068 CNY/t	810-1325 CNY/t	Ref[54]
Grid electricity price[a]	580 CNY/MWh	522-638 CNY/t	Ref[55]
Water electrolyzer cost[b]	3827 CNY/t H_2	3637-3934 CNY/t H_2	Ref[44,56-60]
Battery storage cost[c]	2794 CNY/t H_2	2728-2831 CNY/t H_2	Ref[44,59-61]

Note: (a) We use 90-110% of central estimate of the grid electricity price for low to high estimates.

(b) We use 25 years for alkaline water electrolysis lifetime with a capital cost of 659 USD/kW and an operating cost of 33 USD/kW in 2030[56-58]. We refer to a green hydrogen project that includes 10 MW of water electrolyzers with H_2 production of 936 tonne per year[48]. Using a discount rate of 2.0% (1.5-3.0%)[59] and a USD/CNY exchange rate of 6.8 (the average over 2016-2021)[60], we estimate that 2030 water electrolyzer cost per tonne of H_2 = $(659\times10\times1000+33\times10\times1000\times(1-(\frac{1}{1+2.0\%})^{25})\div(1-\frac{1}{1+2.0\%}))\div(936\times25)\times6.8$ = 3827 CNY/tH_2, with low and high estimates of 3637 and 3934 CNY/tH_2, respectively.

(c) We use 20 years for battery storage lifetime with a capital cost of 566 USD/kW and an operating cost of 14 USD/kW in 2030[61]. We refer to a green hydrogen project that includes 375 MW of battery storage with a four-hour discharge rate for H_2 production of 36500 tonne per year[44]. Using a discount rate of 2.0% (1.5-3.0%)[59] and a USD/CNY exchange rate of 6.8 (the average over 2016-2021)[60], we estimate that 2030 battery storage cost per tonne of H_2 = $(566\times375\times1000+14\times375\times1000\times(1-(\frac{1}{1+2.0\%})^{20})\div(1-\frac{1}{1+2.0\%}))\div(36500\times20)\times6.8$ = 2794 CNY/tH_2, with low and high estimates of 2728 and 2831 CNY/tH_2, respectively.

According to parameters in this table and calculations in Supplementary Table 22, annual cost changes in the MG/HG scenario are -267 billion CNY for feedstock coal, -23 billion CNY for fuel coal, +120 billion CNY for electrolyzers, and +1050 billion CNY for electricity consumption for water electrolysis. Thus, net costs for GHG mitigation in the MG/HG scenarios are 879 billion CNY.

Supplementary Table 21 Cost parameters for provincial solar and wind electricity generation (in 2020 CNY)

Provincial region	Solar electricity cost in 2030 (CNY/MWh)			Wind electricity cost in 2030 (CNY/MWh)		
	Central	Low	High	Central	Low	High
Beijing	111	106	114	120	117	123
Tianjin	115	110	118	129	125	131
Hebei	110	105	113	124	121	127
Shanxi	105	100	107	162	157	165
Inner Mongolia	96	92	99	178	172	181
Liaoning	114	109	117	145	140	147
Jilin	116	110	119	113	110	115
Heilongjiang	119	114	123	117	114	119
Shanghai	123	117	126	111	108	113
Jiangsu	119	114	122	192	186	195
Zhejiang	117	112	120	125	121	127
Anhui	119	114	122	194	188	198
Fujian	107	102	110	131	126	133
Jiangxi	118	112	121	175	170	178
Shandong	116	110	119	131	126	133
Henan	119	114	122	259	251	263
Hubei	123	117	126	156	151	158
Hunan	126	120	129	166	161	169
Guangdong	107	103	110	170	164	172
Guangxi	112	107	115	196	190	199
Hainan	96	91	98	126	122	128
Chongqing	136	130	140	166	161	169
Sichuan	107	102	110	120	117	123
Guizhou	111	106	114	151	147	154
Yunnan	86	82	88	138	134	140
Tibet	67	64	69	296	287	301
Shaanxi	108	103	111	127	123	129
Gansu	96	92	99	166	161	169
Qinghai	79	76	81	180	174	183
Ningxia	96	92	99	132	128	134
Xinjiang	107	102	110	122	118	124

Note:

We use 25 years for solar PV lifetime[62] and 20 years for onshore wind turbine lifetime[63]. We use a capital cost of 392 USD/kW and an operating cost of 17 USD/kW for solar PV in 2030, and a

capital cost of 597 USD/kW and an operating cost of 22 USD/kW for onshore wind turbines in 2030[61,64]. These capital and operating costs in 2030 are estimated using 2021 costs reported by International Renewable Energy Agency[64] and cost reduction rates during 2021-2030 projected by U.S. National Renewable Energy Laboratory[61].

Using a discount rate of 2.0% (1.5-3.0%)[59] and a USD/CNY exchange rate of 6.8 (the average over 2016-2021)[60] and assuming a capacity of 1 MW for solar and wind power facility, we estimate that:

Provincial solar electricity cost in 2030 (CNY/MWh) = $(392\times1000+17\times1000\times(1-(\frac{1}{1+discount\ rate})^{25})\div(1-\frac{1}{1+discount\ rate}))\div(25\times365\times24\times$Provincial solar capacity factor in Supplementary Table 16$)\times6.8$,

and Provincial wind electricity cost in 2030 (CNY/MWh) = $(597\times1000+22\times1000\times(1-(\frac{1}{1+discount\ rate})^{20})\div(1-\frac{1}{1+discount\ rate}))\div(20\times365\times24\times$Provincial wind capacity factor in Supplementary Table 16$)\times6.8$.

Supplementary Table 22 GHG mitigation costs in 2030 for China's coal chemical production relative to the baseline scenario (in 2020 CNY)

	Cost changes in the SE/WE scenario (B CNY)				Cost changes in the SE scenario (B CNY)			Cost changes in the WE scenario (B CNY)			Current cost[g] (CNY/t)	Cost change _SE (CNY/t)[h]	Cost change _WE (CNY/t)[h]
	Feedstock coal[a]	Fuel coal[b]	Electrolyzer[c]	Battery[c]	Electricity for electrolysis[d]	Electricity for other uses[e]	Net change[f]	Electricity for electrolysis[d]	Electricity for other uses[e]	Net change[f]			
Coal-to-oil (DL)	-3.77	-0.25	1.69	1.23	2.46	0.00	1.36	4.53	0.00	3.43	4000[65]	585 (+15%)	1479 (+37%)
Coal-to-oil (IDL)	-27.29	-2.59	12.23	8.93	18.47	-5.39	4.36	27.31	-4.85	13.73	3900[65]	427 (+11%)	1345 (+34%)
Coal-to-natural gas	-43.26	-3.67	19.39	14.15	30.49	-1.90	15.20	43.09	-1.73	27.97	2800[66]	934 (+33%)	1719 (+61%)
Coal-to-olefin	-40.04	-3.85	17.94	13.10	26.81	-14.54	-0.58	41.64	-12.89	15.90	7600[67]	-51 (-0.7%)	1415 (+19%)
Coal-to-ethylene glycol	-17.50	-2.11	7.84	5.72	12.78	-2.27	4.48	19.22	-2.01	11.17	4100[68]	580 (+14%)	1449 (+35%)
Coal-to-coke	0.00	0.00	0.00	0.00	0.00	-10.68	-10.68	0.00	-9.68	-9.68	1600[69]	-27 (-1.7%)	-25 (-1.5%)
Coal-to-calcium carbide	0.00	0.00	0.00	0.00	0.00	-44.90	-44.90	0.00	-40.25	-40.25	2600[70]	-1628 (-63%)	-1459 (-56%)
Coal-to-ammonia	-78.24	-5.27	35.06	25.60	59.90	-17.91	19.13	85.73	-16.05	46.83	2500[71]	425 (+17%)	1041 (+42%)
Coal-to-methanol	-56.94	-5.47	25.52	18.63	41.73	-5.42	18.04	60.31	-4.87	37.18	2500[72]	437 (+17%)	901 (+36%)

Note: DL = direct liquefaction; IDL = indirect liquefaction; SE = Onsite Solar Electricity; and WE = Onsite Wind Electricity.

(a) Cost changes in feedstock coal = -2030 national production of coal chemicals (Supplementary Tables 4-5) × Coal-based H_2 demand per tonne of coal chemicals (= Electrolytic H_2 demand per tonne in Supplementary Table 13) × Feedstock coal needed for H_2 production (Supplementary Table 15) × Feedstock coal price (Supplementary Table 20).

(b) Cost changes in fuel coal = -2030 national production of coal chemicals (Supplementary Tables 4-5) × (Air separation-based O_2 demand per tonne of coal chemicals (Supplementary Table 13) × Coal needed for electricity generation to drive air separation for O_2 production (Supplementary Table 15) + Reductions in coal-based H_2 demand per tonne of coal chemicals (= Electrolytic H_2 demand per tonne in Supplementary Table 13) × Feedstock coal needed for H_2 production (Supplementary Table 15) × Coal needed for electricity generation to drive coal gasification (Supplementary Table 15))× Fuel coal price (Supplementary Table 20).

(c) Cost changes in electrolyzer/battery manufacturing = 2030 national production of coal chemicals (Supplementary Tables 4-5) × Electrolytic H_2 demand per tonne of coal chemicals (Supplementary Table 13) × Electrolyzer/battery cost (Supplementary Table 20).

(d) Cost changes in electricity consumption for water electrolysis in the SE/WE scenario = Σ (2030 provincial production of coal chemicals (Supplementary Table 6) × Electrolytic H_2 demand per tonne of coal chemicals (Supplementary Table 13) × Electricity needed for H_2 production *via* water electrolysis (Supplementary Table 15) × Provincial solar/wind electricity cost (Supplementary Table 21)).

(e) Cost changes in electricity consumption for other uses in the SE/WE scenario = Σ (2030 provincial production of coal chemicals (Supplementary Table 6) × Grid electricity demand per tonne of coal chemicals (Supplementary Table 8) × (Provincial solar/wind electricity cost (Supplementary Table 21) – Grid electricity price (Supplementary Table 20))).

(f) Net change is the sum of all the cost changes.

(g) We collect up-to-date production costs for coal chemicals. We use the 2030 national production of coal chemicals (Supplementary Tables 4-5) and these production costs to estimate that total production cost of coal chemicals in 2030 is 1131 billion CNY.

(h) Cost changes per tonne of coal chemicals = Net cost changes of coal chemicals in the SE/WE scenario ÷ 2030 national production of coal chemicals (Supplementary Tables 4-5). Numbers in parentheses refer to cost change rates relative to current production costs.

Supplementary Table 23 Provincial options for solar or wind-based water electrolysis to achieve the maximum GHG mitigation/minimum cost of national coal chemical production

Provincial region	Solution for maximum national GHG mitigation			Solution for minimum national mitigation cost		
	Provincial option	GHG mitigation ($MtCO_2eq$)	Cost change (B CNY)	Provincial option	GHG mitigation ($MtCO_2eq$)	Cost change (B CNY)
Beijing	-	-1.01	0.00	-	-1.01	0.00
Tianjin	Wind	-2.85	0.21	Solar	-2.42	0.15
Hebei	Wind	-17.84	0.93	Solar	-14.76	0.32
Shanxi	Wind	-54.95	6.48	Solar	-54.69	0.17
Inner Mongolia	Solar	-145.62	-7.39	Solar	-145.62	-7.39
Liaoning	Solar	-20.48	2.65	Solar	-20.48	2.65
Jilin	Solar	-3.23	0.09	Wind	-3.15	0.08
Heilongjiang	Solar	-6.12	0.21	Wind	-5.95	0.19
Shanghai	Wind	-4.46	0.32	Wind	-4.46	0.32
Jiangsu	Wind	-20.70	4.55	Solar	5.57	1.50
Zhejiang	Wind	-7.93	0.38	Solar	0.52	0.31
Anhui	Wind	-23.97	6.90	Solar	-18.71	2.02
Fujian	Wind	-6.67	0.58	Solar	-6.22	0.31
Jiangxi	Wind	-3.00	-0.41	Solar	2.52	-0.54
Shandong	Solar	-54.47	5.65	Solar	-54.47	5.65
Henan	Solar	-38.39	3.96	Solar	-38.39	3.96
Hubei	Solar	-17.32	1.87	Solar	-17.32	1.87
Hunan	Solar	-5.10	0.22	Solar	-5.10	0.22
Guangdong	Wind	-8.38	-0.11	Solar	-6.18	-0.13
Guangxi	Solar	-5.67	0.22	Solar	-5.67	0.22
Hainan	Solar	-6.59	0.59	Solar	-6.59	0.59
Chongqing	Solar	-12.53	2.26	Solar	-12.53	2.26
Sichuan	Wind	-12.14	-0.07	Solar	-10.16	-0.68
Guizhou	Solar	-14.01	1.01	Solar	-14.01	1.01
Yunnan	Solar	-9.66	-0.99	Solar	-9.66	-0.99
Tibet	-	-0.01	0.00	-	-0.01	0.00
Shaanxi	Wind	-69.31	3.00	Solar	-66.34	-0.87
Gansu	Solar	-5.18	-1.32	Solar	-5.18	-1.32
Qinghai	Solar	-11.62	-0.99	Solar	-11.62	-0.99
Ningxia	Solar	-58.38	-3.97	Solar	-58.38	-3.97
Xinjiang	Solar	-74.57	-0.67	Solar	-74.57	-0.67
Total	-	-722.15	26.17	-	-665.04	6.26

Note: Solar/wind refers to solar/wind-based water electrolysis. Beijing and Tibet have no coal chemical production. GHG mitigation and costs are calculated based on central estimates of cost parameters in Supplementary Tables 20-21. GHG mitigation costs per tonne in solutions for the maximum national GHG mitigation and minimum national mitigation cost are 36 and 9.4 CNY/tCO_2eq, respectively.

Supplementary Table 24 Excess O_2 calculations

Coal chemical	Electrolytic O_2 produced with electrolytic H_2 (tO_2/t product)	Electrolytic O_2 demand (tO_2/t product)	Excess electrolytic O_2 (tO_2/t product)	Excess electrolytic O_2 (MtO_2)
Coal-to-oil (direct liquefaction)	1.52	0.00	1.52	3.53
Coal-to-oil (indirect liquefaction)	2.50	0.92	1.59	16.20
Coal-to-natural gas	2.49	0.58	1.91	31.14
Coal-to-olefin	3.34	1.27	2.06	23.18
Coal-to-ethylene glycol	2.12	1.50	0.62	4.80
Coal-to-coke	0.00	0.00	0.00	0.00
Coal-to-calcium carbide	0.00	0.00	0.00	0.00
Coal-to-ammonia	1.63	0.00	1.63	73.29
Coal-to-methanol	1.29	0.49	0.80	32.97
Total	-	-	-	185.12

Note: Electrolytic O_2 produced with electrolytic H_2 per tonne of coal chemicals = Electrolytic H_2 demand per tonne of coal chemicals (Supplementary Table 13) ÷ 2 (molecular weight of H_2) × 16 (relative atomic mass of O).

Electrolytic O_2 demands per tonne of coal chemicals are calculated as in Supplementary Table 13.

Excess electrolytic O_2 per tonne of coal chemicals = Electrolytic O_2 produced with electrolytic H_2 per tonne of coal chemicals – Electrolytic O_2 demand per tonne of coal chemicals.

Excess electrolytic O_2 = 2030 national production of coal chemicals (Supplementary Tables 4-5) × Excess electrolytic O_2 per tonne of coal chemicals.

We assume the selling price of electrolytic O_2 to be equal to the air separation-based O_2 production cost (364 CNY/tO_2)[33]. Since electrolytic O_2 is a by-product of electrolytic H_2, using electrolytic O_2 can reduce 0.26 tCO_2eq/t O_2 from coal-driven air separation (life-cycle GHG emission factor of air separation-based O_2 production in Supplementary Table 15). Thus, using 185 Mt of excess electrolytic O_2 brings GHG mitigation of 48 $MtCO_2$eq.

Supplementary Table 25 Land area needed for renewable electricity generation in the Onsite Solar Electricity (SE) and Onsite Wind Electricity (WE) Scenarios

Provincial region	Renewable electricity needed (TWh)[a]	Renewable capacity needed (GW)[b]		Land conversion factor (km^2/GW)		Land area needed (1000 km^2)[e]	
		Solar	Wind	Solar[c]	Wind[d]	Solar	Wind
Beijing	0.00	0.00	0.00	27.50	73.10	0.00	0.00
Tianjin	4.36	2.52	1.71	26.06	73.10	0.07	0.13
Hebei	42.12	23.29	15.98	26.32	73.10	0.61	1.17
Shanxi	109.41	57.63	54.17	23.98	73.10	1.38	3.96
Inner Mongolia	438.26	212.14	237.25	25.02	73.10	5.31	17.34
Liaoning	58.92	33.71	26.00	29.20	73.10	0.98	1.90
Jilin	4.93	2.87	1.70	32.61	73.10	0.09	0.12
Heilongjiang	10.94	6.57	3.91	49.11	73.10	0.32	0.29
Shanghai	9.44	5.83	3.20	19.22	73.10	0.11	0.23
Jiangsu	42.16	25.29	24.63	20.56	73.10	0.52	1.80
Zhejiang	8.99	5.29	3.43	18.16	73.10	0.10	0.25
Anhui	64.80	38.78	38.41	19.29	73.10	0.75	2.81
Fujian	11.46	6.16	4.56	16.35	73.10	0.10	0.33
Jiangxi	2.29	1.35	1.22	17.18	73.10	0.02	0.09
Shandong	146.45	85.19	58.29	22.94	73.10	1.95	4.26
Henan	117.73	70.54	92.82	20.56	73.10	1.45	6.79
Hubei	57.12	35.24	27.12	19.29	73.10	0.68	1.98
Hunan	11.47	7.25	5.82	17.18	73.10	0.12	0.43
Guangdong	0.36	0.19	0.18	15.29	73.10	0.00	0.01
Guangxi	11.95	6.73	7.13	15.60	73.10	0.11	0.52
Hainan	21.86	10.50	8.41	14.36	73.10	0.15	0.61
Chongqing	40.68	27.88	20.65	18.66	73.10	0.52	1.51
Sichuan	45.64	24.61	16.77	18.76	73.10	0.46	1.23
Guizhou	32.19	17.94	14.85	17.18	73.10	0.31	1.09
Yunnan	30.83	13.37	12.97	16.41	73.10	0.22	0.95
Tibet	0.00	0.00	0.00	19.39	73.10	0.00	0.00
Shaanxi	206.95	112.56	80.02	22.34	73.10	2.51	5.85
Gansu	11.72	5.67	5.95	24.29	73.10	0.14	0.43
Qinghai	42.93	17.11	23.55	22.34	73.10	0.38	1.72
Ningxia	204.42	99.13	82.17	23.98	73.10	2.38	6.01
Xinjiang	236.55	127.33	88.11	33.31	73.10	4.24	6.44
Total	2026.90	1082.69	961.02	-	-	26.00	70.25

Note: (a) Provincial renewable electricity needed in the SE/WE scenario = 2030 provincial production of coal chemicals (Supplementary Table 6) × Electrolytic H_2 demand per tonne of coal

chemicals (Supplementary Table 13) × Electricity needed for H_2 production *via* water electrolysis (Supplementary Table 15);

(b) Provincial solar/wind capacity needed in the SE/WE scenario (GW) = Provincial renewable electricity needed (TWh) ÷ (365 × 24 × Provincial solar/wind capacity factor in Supplementary Table 16) × 1000;

(c) To determine land area needed for solar power deployment, we use a land conversion factor that considers the latitudes of provincial regions. Land conversion factors for various latitudes are obtained from guidelines released by the Ministry of Land and Resources of China[73] and aggregated by Yang et al[74]. At a lower latitude, the sun is higher and PV arrays cast shorter shadows, resulting in smaller land area needed for PV installations of a given capacity than that needed at a higher latitude[74]. Thus, we identify latitude ranges of provincial regions and assign suitable land conversion factors to provincial regions.

(d) A designed distance between wind turbines, according to national guidelines, is five times the rotor diameter (5D) in the direction of the prevailing wind and 3D across the prevailing wind[75]. As 6 MW wind turbines tend to be widely used in the near future, we select a typical Goldwind 171-6.0MW for our analysis, of which the rotor diameter is 171 m[76]. Thus, each wind turbine occupies 5×0.171×3×0.171 =0.44 km^2, i.e. 73.1 km^2/GW for land conversion factors of onshore wind power.

(e) Provincial land area needed for solar/wind power deployment = Solar/wind power capacity needed ÷ Land conversion factor of solar/wind power.

Supplementary References

1. National Bureau of Statistics of China. *China Statistical Yearbook 2021*. http://www.stats.gov.cn/tjsj/ndsj/2021/indexch.htm (2021).

2. State Council of China. *China Economic Census Yearbook 2018*. http://www.stats.gov.cn/tjsj/pcsj/jjpc/4jp/indexch.htm (2020).

3. National Bureau of Statistics of China. *National statistical data*. http://data.stats.gov.cn/easyquery.htm (2016).

4. Zhang, Y. et al. Intensive carbon dioxide emission of coal chemical industry in China. *Applied Energy* **236**, 540-550 (2019).

5. *Report of China's calcium carbide industry: Market analyses and investment strategies*. https://www.chyxx.com/research/202102/929274.html (2022).

6. *Status of China's ammonia industry development*. http://www.cnfia.com/c/cn/news/2020-08/07/news_12627.html (2020).

7. *Status of China's methonal industry development*. https://cn.agropages.com/News/print-22882.htm (2021).

8. *Analysis of China's methonal supply and demand in 2022*. https://www.qianzhan.com/analyst/detail/220/220128-f0c04087.html (2022).

9. China Coal Processing & Utilization Association, 2020. *China coal deep processing industry development report 2019-2020*. http://www.cciep.net/qkbg/1.html (2020).

10. Chen, Y. & Yang, Q. Research on high-quality development of modern coal chemical industry under background of "double carbon". *Coal Processing & Comprehensive Utilization* **1**, 50-54 (2022).

11. Jin, L. et al. Pathway of carbon emissions peak of China's coal chemical industry. *Research of Environmental Sciences* **35**, 368-376 (2022).

12. Orient Securities. *Analyisis report of China's calcium carbide supply and demand*. https://www.vzkoo.com/read/2022072061bb7950367bc32493438586.html (2022).

13. Liu, X. et al. CO_2 emissions in calcium carbide industry: An analysis of China's mitigation potential. *International Journal of Greenhouse Gas Control* **5**, 1240-1249 (2011).

14. IKE Environmental Technology, Sichuan University. *Chinese Life Cycle Database V0.8*. http://www.ike-global.com/archives/1094.html (2014).

15. Liu, X., Wang, H. & Chen, J. Method and basic model for development of Chinese reference life cycle database of fundamental industries. *Acta Scientiae Circumstantiae* **30**, 2136-2144 (2010).

16. National Energy Administration of China. *Plans for China's coal deep processing*

industry demonstration. http://zfxxgk.nea.gov.cn/auto83/201703/W020170303357509200744.pdf (2017).

17. Cinda Securities. *Financial analysis report of a coal chemical company in China*. https://pdf.dfcfw.com/pdf/H3_AP202011181430589743_1.pdf?1605720153000.pdf (2020).

18. PingAn Securities. *Financial analyses of coal chemical industry in China*. https://www.hstong.com/news/detail/22030213222622497 (2022).

19. ZhongTai Securities. *Analysis report of coal-to-coke industry in China*. http://pdf.dfcfw.com/pdf/H3_AP202006151384987522_1.pdf (2020).

20. Shanghai Brilliance Credit Rating & Investors Service. *Impacts of electricity price adjustment on the PVC industry*. http://www.shxsj.com/uploadfile/kanwu/1231-6.pdf (2015).

21. LeadLeo. *Status analysis of China's ammonia industry in 2019*. http://pdf.dfcfw.com/pdf/H3_AP202008191399559082_1.pdf (2019).

22. Sealand Securities. *Financial analyses of a coal chemical company in China*. https://pdf.dfcfw.com/pdf/H3_AP202206191573190239_1.pdf (2022).

23. World Resources Institute. *Greenhouse gas protocol tool for energy consumption in China V2.1*. http://www.ghgprotocol.org/calculation-tools (2013).

24. Ministry of Ecology and Environment of China. *Guidelines for greenhouse gas emission accounting and reporting of power companies*. https://www.mee.gov.cn/xxgk2018/xxgk/xxgk06/202112/W020211202787053248513.pdf (2021).

25. National Development and Reform Commission of China. *Guidelines for greenhouse gas emission accounting and reporting of chemical companies*. http://www.gov.cn/gzdt/att/att/site1/20131104/001e3741a2cc13e13f1c04.pdf (2013).

26. Zhu, X., Wang, S. & Wang, L. Life cycle analysis of greenhouse gas emissions of China's power generation on spatial and temporal scale. *Energy Science & Engineering* **10**, 1083-1095 (2022).

27. Xu, K. et al. A comprehensive estimate of life cycle greenhouse gas emissions from onshore wind energy in China. *Journal of Cleaner Production* **338**, 130683 (2022).

28. *Efficiency benchmark for China's coal power plants in 2025*. https://coal.in-en.com/html/coal-2607810.shtml (2021).

29. China Electricity Council. *Annual report of China's electricity industry development 2020*. https://cec.org.cn/detail/index.html?3-298428 (2021).

30. International Energy Agency. *World Energy Outlook 2017*. https://www.iea.org/reports/world-energy-outlook-2017 (2017).

31. Shu, Y. et al. Carbon peak and carbon neutrality path for China's power industry. *Strategic Study of Chinese Academy of Engineering* **23**, 1-14 (2021).

32. Wikipedia. *Fischer–Tropsch process*. https://en.wikipedia.org/wiki/Fischer%E2%80%93Tropsch_process (2022).

33. Yu, B., Cao, C. & Gu, W. Energy consumption structure in China and the economic analysis of coupled wind power and coal to substitute natural gas. *Strategic Study of Chinese Academy of Engineering* **17**, 100-106 (2015).

34. Zheng, Z. Features of Shell coal gasification technology. *Coal Chemical Industry* **2**, 7-11 (2003).

35. National Bureau of Statistics of China. *China Energy Statistical Yearbook 2021*. https://data.cnki.net/v3/trade/Yearbook/Single/N2022060061?zcode=Z024 (2021).

36. *Statistics on photovoltaic cell production of China's provincial regions in 2018*. https://msolar.in-en.com/html/solar-2329578.shtml (2019).

37. Chen, G., Yang, Q. & Zhao, Y. Renewability of wind power in China: A case study of nonrenewable energy cost and greenhouse gas emission by a plant in Guangxi. *Renewable and Sustainable Energy Reviews* **15**, 2322-2329 (2011).

38. Qin, Y. et al. Air quality-carbon-water synergies and trade-offs in China's natural gas industry. *Nature Sustainability* **1**, 505-511 (2018).

39. Zhou, M. et al. Environmental benefits and household costs of clean heating options in northern China. *Nature Sustainability* **5**, 329-338 (2022).

40. Huang, G., Li, J., Wei, S., Yang, Y. & Zhou, X. Status and economic analysis of hydrogen production technology from fossil raw materials. *Chemical Industry and Engineering Progress* **38**, 5217-5224 (2019).

41. China National Institute of Standardization, China Hydrogen Technology Standardization Committee (SAC/TC309). *China's hydrogen industry infrastructure development: Pathway to low-carbon and cost-competitive hydrogen*. China Quality and Standards Publishing: Beijing (2019).

42. Cheng, W., Li, J., Liu, H. & Tian, Y. Analysis of life cycle cost of coal hydrogen production chain based on two technical routes. *Coal Economic Research* **40**, 4-11 (2020).

43. The Comprehensive Research Group for Energy Consulting and Research. Strategic research on clean, efficient, sustainable exploitation and utilization of coal in China. *Strategic Study of Chinese Academy of Engineering* **17** (9), 1-5 (2015).

44. Palmer, G., Roberts, A., Hoadley, A., Dargaville, R. & Honnery, D. Life-cycle greenhouse gas emissions and net energy assessment of large-scale hydrogen production via electrolysis and solar PV. *Energy & Environmental Science* **14**, 5113-5131 (2021).

45. Cooper, J., Dubey, L., Bakkaloglu, S. & Hawkes, A. Hydrogen emissions from the

hydrogen value chain-emissions profile and impact to global warming. *Science of The Total Environment* **830**, 154624 (2022).

46. Rozendal, R. A., Hamelers, H. V., Euverink, G. J., Metz, S. J. & Buisman, C. J. Principle and perspectives of hydrogen production through biocatalyzed electrolysis. *International Journal of Hydrogen Energy* **31**, 1632-1640 (2006).

47. UK Department for Energy Security and Net Zero and UK Department for Business, Energy & Industrial Strategy. *Atmospheric implications of increased hydrogen use*. https://www.gov.uk/government/publications/atmospheric-implications-of-increased-hydrogen-use (2022).

48. Toshiba. *The world's largest-class hydrogen production, Fukushima Hydrogen Energy Research Field now is completed at Namie town in Fukushima*. https://www.global.toshiba/ww/news/energy/2020/03/news-20200307-01.html (2020).

49. He, G. & Kammen, D. M. Where, when and how much solar is available? A provincial-scale solar resource assessment for China. *Renewable Energy* **85**, 74-82 (2016).

50. He, G. et al. Rapid cost decrease of renewables and storage accelerates the decarbonization of China's power system. *Nature Communications* **11**, 2486 (2020).

51. Jung, C. & Schindler, D. Development of onshore wind turbine fleet counteracts climate change-induced reduction in global capacity factor. *Nature Energy* **7**, 608-619 (2022).

52. *A rise in PV efficiency is expected by technical innovation*. https://solar.ofweek.com/2021-03/ART-260006-8440-30489446.html (2021).

53. National Development and Reform Commission of China. *Improvement measures for the coal market price formation mechanism*. https://www.ndrc.gov.cn/xwdt/tzgg/202202/t20220225_1317006_ext.html (2022).

54. *Analysis of revenues from coal for coal chemical production*. https://www.jiemian.com/article/7508770.html (2022).

55. *Summary of grid electricity prices in China (2018)*. https://www.sohu.com/a/271827080_146940 (2018).

56. Yang, X., Nielsen, C. P., Song, S. & McElroy, M. B. Breaking the hard-to-abate bottleneck in China's path to carbon neutrality with clean hydrogen. *Nature Energy* **7**, 955-965 (2022).

57. Sgobbi, A. et al. How far away is hydrogen? Its role in the medium and long-term decarbonisation of the European energy system. *International Journal of Hydrogen Energy* **41**, 19-35 (2016).

58. Parkinson, B., Balcombe, P., Speirs, J., Hawkes, A. & Hellgardt, K. Levelized cost of CO_2 mitigation from hydrogen production routes. *Energy & Environmental Science* **12**, 19-40 (2019).

59. Rennert, K. et al. Comprehensive evidence implies a higher social cost of CO_2. *Nature* **610**, 687-692 (2022).

60. U.S. Internal Revenue Service. *Yearly Average Currency Exchange Rates*. https://www.irs.gov/individuals/international-taxpayers/yearly-average-currency-exchange-rates (2022).

61. U.S. National Renewable Energy Laboratory. *Electricity Annual Technology Baseline Data*. https://atb.nrel.gov/electricity/2021/data (2021).

62. Chowdhury, M. S. et al. An overview of solar photovoltaic panels' end-of-life material recycling. *Energy Strategy Reviews* **27**, 100431 (2020).

63. Xu, L., Pang, M., Zhang, L., Poganietz, W. R. & Marathe, S. D. Life cycle assessment of onshore wind power systems in China. *Resources, Conservation and Recycling* **132**, 361-368 (2018).

64. International Renewable Energy Agency. *Renewable power generation costs in 2021*. https://irena.org/publications/2022/Jul/Renewable-Power-Generation-Costs-in-2021 (2021).

65. *Analysis of coal-to-oil production costs and revenues*. https://www.meitanwang.com/meitan/b5/664688.html (2014).

66. *Coal-to-natural gas projects started to deploy extensively in China*. https://www.hxny.com/nd-71607-0-47.html (2022).

67. National Development and Reform Commission of China. *Coal chemical industry is cost-competitive under a high oil price*. https://www.ndrc.gov.cn/wsdwhfz/202204/t20220429_1324160.html (2022).

68. Huang, P. *Cost-competitiveness analysis of coal-to-ethylene glycol in China*. http://www.pmweb.com.cn/news/1453.html (2020).

69. Everbright Securities. *Financial analysis report of a coal-to-coke company in China*. https://pdf.dfcfw.com/pdf/H3_AP201909231365571585_1.pdf?1601214828000.pdf (2019).

70. ZhongTai Securities. *Financial analysis report of China's calcium carbide industry*. http://pdf.dfcfw.com/pdf/H3_AP202007061389795054_1.pdf (2020).

71. Li, J. & Wang, M. Economic analysis of ammonia mixed coal-fired power generation technology based on different scenario modes. *China Coal* **48**, 54-59 (2022).

72. Cinda Securities. *Financial analysis report of China's coal-to-methonal industry*. https://pdf.dfcfw.com/pdf/H3_AP202206161572489065_1.pdf?1655378090000.pdf (2022).

73. Ministry of Land and Resources of China. *Land use control index for PV station project*. http://g.mnr.gov.cn/201701/t20170123_1429897.html (2015).

74. Yang, Q. et al. A GIS-based high spatial resolution assessment of large-scale PV generation potential in China. *Applied energy* **247**, 254-269 (2019).

75. National Development and Reform Commission of China. *Technical specifications of wind power plant design (DL/T 5383-2007)*. (2007).

76. *The first commercialized 6MW onshore wind turbine in China was installed successfully*. https://www.china5e.com/news/news-1127442-1.html (2021).
