# Supplementary material for: Deploying green hydrogen to decarbonize China’s coal chemical sector
Source: Nat Commun. 2023 Dec 7;14:8104. doi: 10.1038/s41467-023-43540-4 (PMC10703803; doi:10.1038/s41467-023-43540-4)
Supplement: Supplementary file 3 — Reporting Summary [file 41467_2023_43540_MOESM3_ESM.pdf]

nature research

Corresponding author(s): Yang Guo
Denise L. Mauzerall

Last updated by author(s): Oct 26, 2023

Reporting Summary

Nature Research wishes to improve the reproducibility of the work that we publish. This form provides structure for consistency and transparency in reporting. For further information on Nature Research policies, see our Editorial Policies and the Editorial Policy Checklist.

Statistics

For all statistical analyses, confirm that the following items are present in the figure legend, table legend, main text, or Methods section.

n/a	Confirmed	
☒	☐	The exact sample size (*n*) for each experimental group/condition, given as a discrete number and unit of measurement
☒	☐	A statement on whether measurements were taken from distinct samples or whether the same sample was measured repeatedly
☒	☐	The statistical test(s) used AND whether they are one- or two-sided<br>*Only common tests should be described solely by name; describe more complex techniques in the Methods section.*
☒	☐	A description of all covariates tested
☒	☐	A description of any assumptions or corrections, such as tests of normality and adjustment for multiple comparisons
☐	☒	A full description of the statistical parameters including central tendency (e.g. means) or other basic estimates (e.g. regression coefficient) AND variation (e.g. standard deviation) or associated estimates of uncertainty (e.g. confidence intervals)
☒	☐	For null hypothesis testing, the test statistic (e.g. F, t, r) with confidence intervals, effect sizes, degrees of freedom and P value noted<br>*Give P values as exact values whenever suitable.*
☒	☐	For Bayesian analysis, information on the choice of priors and Markov chain Monte Carlo settings
☒	☐	For hierarchical and complex designs, identification of the appropriate level for tests and full reporting of outcomes
☒	☐	Estimates of effect sizes (e.g. Cohen's d, Pearson's r), indicating how they were calculated

Our web collection on statistics for biologists contains articles on many of the points above.

Software and code

Policy information about availability of computer code

Data collection	No software was used for data collection.
Data analysis	We conduct data analyses and presentation using Excel 2023 and ArcGIS V10.4.1.

For manuscripts utilizing custom algorithms or software that are central to the research but not yet described in published literature, software must be made available to editors and reviewers. We strongly encourage code deposition in a community repository (e.g. GitHub). See the Nature Research guidelines for submitting code & software for further information.

Data

Policy information about availability of data

All manuscripts must include a data availability statement. This statement should provide the following information, where applicable:

- Accession codes, unique identifiers, or web links for publicly available datasets
- A list of figures that have associated raw data
- A description of any restrictions on data availability

The source data that support all figures in the main text and Supplementary Information are provided as the Source Data file. All data used for this study are provided in Source Data and Supplementary Information. We confirm that all data and calculations needed for our study are published as supplementary files with the article.

Field-specific reporting

Please select the one below that is the best fit for your research. If you are not sure, read the appropriate sections before making your selection.

☐ Life sciences ☐ Behavioural & social sciences ☒ Ecological, evolutionary & environmental sciences

For a reference copy of the document with all sections, see nature.com/documents/nr-reporting-summary-flat.pdf

Ecological, evolutionary & environmental sciences study design

All studies must disclose on these points even when the disclosure is negative.

Study description	This study explores the GHG mitigation potential and costs for onsite deployment of green hydrogen and oxygen in China's coal chemical sector, using a life-cycle assessment and techno-economic analyses.
Research sample	31 provincial regions (except for Hong Kong, Macau, and Taiwan where no coal chemical data are available) in China
Sampling strategy	No sampling strategy
Data collection	We integrate multiple data sources to collect the data used for this study, including 1) statistics and reports of coal chemical industry, 2) national statistic yearbooks and reports, 3) government documents, 4) technical project reports, 5) financial analysis reports, 6) existing life-cycle databases, and 7) previous studies.<br>We refer to all data sources throughout the main text and Supplementary Information.
Timing and spatial scale	2020-2030 in China
Data exclusions	No data exclusion
Reproducibility	All data used for this study are provided in Source Data and Supplementary Information. We formulate our modeling into calculations step by step for each parameter and result. All calculations and formulations in this study are detailed in the Supplementary Information for reproductivity. We confirm that all data and calculations needed for our study are published as supplementary files with the article.
Randomization	The data are not from a random sampling, and randomization is not involved.
Blinding	Blinding methods are not involved in this study.

Did the study involve field work? ☐ Yes ☒ No

Reporting for specific materials, systems and methods

We require information from authors about some types of materials, experimental systems and methods used in many studies. Here, indicate whether each material, system or method listed is relevant to your study. If you are not sure if a list item applies to your research, read the appropriate section before selecting a response.

Materials & experimental systems

n/a	Involved in the study	
☒	☐	Antibodies
☒	☐	Eukaryotic cell lines
☒	☐	Palaeontology and archaeology
☒	☐	Animals and other organisms
☒	☐	Human research participants
☒	☐	Clinical data
☒	☐	Dual use research of concern

Methods

n/a	Involved in the study	
☒	☐	ChIP-seq
☒	☐	Flow cytometry
☒	☐	MRI-based neuroimaging
